# A unique clade of light-driven proton-pumping rhodopsins evolved in the cyanobacterial lineage

**DOI:** 10.1038/s41598-020-73606-y

**Published:** 2020-10-07

**Authors:** Masumi Hasegawa, Toshiaki Hosaka, Keiichi Kojima, Yosuke Nishimura, Yu Nakajima, Tomomi Kimura-Someya, Mikako Shirouzu, Yuki Sudo, Susumu Yoshizawa

**Affiliations:** 1 Atmosphere and Ocean Research Institute, The University of Tokyo, Chiba, 277-8564 Japan; 2 Graduate School of Frontier Sciences, The University of Tokyo, Chiba, 277-8563 Japan; 3 Laboratory for Protein Functional and Structural Biology, RIKEN Center for Biosystems Dynamics Research, Kanagawa, 230-0045 Japan; 4 Graduate School of Medicine, Dentistry and Pharmaceutical Sciences, Okayama University, Okayama, 700-8530 Japan; 5 Bioproduction Research Institute, National Institute of Advanced Industrial Science and Technology, Ibaraki, 305-8766 Japan; 6 Collaborative Research Institute for Innovative Microbiology, The University of Tokyo, Tokyo, 113-8657 Japan

**Keywords:** Microbial ecology, Membrane biophysics, Bacterial genomics

## Abstract

Microbial rhodopsin is a photoreceptor protein found in various bacteria and archaea, and it is considered to be a light-utilization device unique to heterotrophs. Recent studies have shown that several cyanobacterial genomes also include genes that encode rhodopsins, indicating that these auxiliary light-utilizing proteins may have evolved within photoautotroph lineages. To explore this possibility, we performed a large-scale genomic survey to clarify the distribution of rhodopsin and its phylogeny. Our surveys revealed a novel rhodopsin clade, cyanorhodopsin (CyR), that is unique to cyanobacteria. Genomic analysis revealed that rhodopsin genes show a habitat-biased distribution in cyanobacterial taxa, and that the CyR clade is composed exclusively of non-marine cyanobacterial strains. Functional analysis using a heterologous expression system revealed that CyRs function as light-driven outward H^+^ pumps. Examination of the photochemical properties and crystal structure (2.65 Å resolution) of a representative CyR protein, N2098R from *Calothrix* sp. NIES-2098, revealed that the structure of the protein is very similar to that of other rhodopsins such as bacteriorhodopsin, but that its retinal configuration and spectroscopic characteristics (absorption maximum and photocycle) are distinct from those of bacteriorhodopsin. These results suggest that the CyR clade proteins evolved together with chlorophyll-based photosynthesis systems and may have been optimized for the cyanobacterial environment.

## Introduction

Energy derived from sunlight drives almost all biological processes on earth. The representative organisms that channel light energy from the sun into ecosystems are photoautotrophs, such as cyanobacteria, which perform photosynthesis using chlorophyll. In addition, cyanobacteria have adapted to use fluctuating light efficiently in their habitats through the evolution of photoacclimation processes^1,2^. These processes are known to involve a variety of antenna pigments to harvest light that chlorophyll cannot directly absorb and transfer light energy to the reaction center of photosynthesis. However, recent culture-independent surveys have shown that a diverse range of bacterial taxa, including several strains of cyanobacteria, have microbial rhodopsin (hereafter rhodopsin), which is a light-energy-harvesting device distinct from chlorophyll-based photosystems. This raises the possibility that rhodopsin-mediated photosystems have evolved in the cyanobacterial lineage.

Rhodopsins are seven-transmembrane proteins that contain retinal as a light-absorbing chromophore and a light-induced *trans*–*cis* isomerization of retinal triggers their activities. Various functions of rhodopsins have been found in prokaryotes, but they can be broadly separated into two types: (i) light sensors that work as phototaxis receptors and enzymes (e.g., sensory rhodopsin-I and -II in the archaeon *Halobacterium salinarum*^3^) and (ii) ion pumps that use light energy to create an ion-motive force that can be used by cells as a type of intracellular energy. Bacteriorhodopsin (BR), the first ion pumping rhodopsin, functions as a light-driven H^+^ pump^4^. Later, halorhodopsin (HR), a light-driven inward Cl^−^ pump, was discovered from the same species^5^. Although the sensory rhodopsins appear to be restricted to hypersaline environments, ion-pumping rhodopsins have been found from a vast range of environments; for example, proteorhodopsin (PR; H^+^ pump) and Na^+^-pumping rhodopsin (NaR; Na^+^ pump) have been found in marine microbes^6,7^, DTG-motif rhodopsin (H^+^ pump) has been found in terrestrial microbes^8^, and xanthorhodopsin (H^+^ pump) has been found in microbes colonizing hypersaline environments^9^. The discovery of ion-pumping rhodopsins in such a diverse range of environments suggests that the conversion of sunlight to a bioavailable ion-motive force has been a crucial part of the adaptation strategies of a variety of microbes^10,11^.

Rhodopsin genes have been found in many heterotrophic bacteria and archaea, and so these microbes can also be classified as photoheterotrophs. However, several photoautotrophic cyanobacteria have also been reported to possess rhodopsin genes. In 2003, a rhodopsin gene was found in the cyanobacteria *Anabaena* sp. PCC 7120 and the protein it encodes was named *Anabaena* sensory rhodopsin (ASR) because its amino acid sequence lacks a proton donor and it cannot detectably transport ions^12^. Subsequent studies have shown that ASR is included in the xenorhodopsin (XeR; light-driven inward H^+^ pump) clade, although the intracellular biological role of the rhodopsins in this clade remains unclear^13^. Next, a xanthorhodopsin-like rhodopsin (XLR), *Gloeobacter* rhodopsin (GR; light-driven outward H^+^ pump) was found in the cyanobacterium *Gloeobacter violaceus* PCC 7421^14–16^. In addition, the cyanobacterial halorhodopsin (CyHR; light-driven inward Cl^−^ pump) was recently found in *Mastigocladopsis repens* PCC 10914 and *Synechocystis* sp. PCC 7509^17,18^. Most of the genes in the CyHR clade are possessed only by cyanobacteria; however, they have also been found in a few marine bacteria^19^. Together, this fragmented evidence implies that rhodopsin-mediated photosystems may have evolved in the cyanobacterial lineage together with chlorophyll-based photosynthesis systems to allow for more efficient utilization of solar energy, although the distribution of rhodopsin genes in the cyanobacterial lineage remains to be clarified^20^.

Here, we performed a comprehensive homology search for rhodopsin genes in 154 cyanobacterial genomes to clarify the distribution of rhodopsin genes within the cyanobacterial lineage. Phylogenetic analysis based on the detected rhodopsin genes revealed an ecologically biased distribution of rhodopsin genes in the cyanobacterial lineage as well as a cyanobacteria-specific rhodopsin clade containing hitherto unknown rhodopsins. Heterologous expression analysis was used to examine the function of the rhodopsins in this novel cyanobacteria-specific clade, and spectroscopic and X-ray structural analyses were performed to investigate the possibility that the photochemical properties of these rhodopsins are tuned specifically for the cyanobacterial environment.

## Results

### Phylogenetic analysis and distribution of rhodopsin genes in cyanobacteria

**Figure 1:**
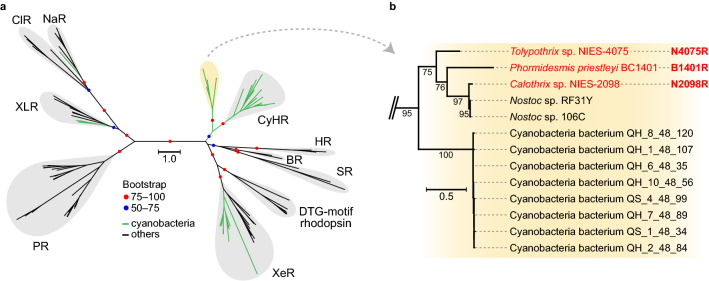
The novel cyanorhodopsin clade. (**a**) Maximum likelihood tree of amino acid sequences of microbial rhodopsins. Bootstrap probabilities (≥ 50%) are indicated by colored circles. Green branches indicate cyanobacterial rhodopsins, and black branches indicate others. Rhodopsin clades are as follows: NaR (Na^+^-pumping rhodopsin), ClR (Cl^−^-pumping rhodopsin), XLR (xanthorhodopsin-like rhodopsin), PR (proteorhodopsin), XeR (xenorhodopsin), DTG-motif rhodopsin, SR (sensory rhodopsin-I and sensory rhodopsin-II), BR (bacteriorhodopsin), HR (halorhodopsin), CyHR (cyanobacterial halorhodopsin), and a novel cyanobacteria-specific clade (yellow shading). (**b**) Enlarged view of the novel cyanobacteria-specific clade. The three rhodopsins functionally examined in this study are shown in red. The scale bar represents substitutions per site.

To survey the distribution of rhodopsin genes in cyanobacteria, a sequence homology search was performed using 154 cyanobacterial genomes, including 126 genomes used in a large-scale comparative genomic study of cyanobacteria^21^ and 28 genomes known to possess rhodopsin genes obtained from a public database. Based on this search, 56 rhodopsin genes in 42 cyanobacterial genomes were identified (Table 1). Stratified by the habitat, the rhodopsin genes were found almost exclusively in freshwater cyanobacteria: 29 in freshwater, 9 in high salinity, 2 in marine, and 2 in NA (not available). There are, however, 9 genomes from a high salinity environment, 8 were from the same site and showed little genetic variation. Phylogenetic analysis of the amino acid sequences of the rhodopsins encoded by the 56 identified genes revealed that the proteins belonged to four known rhodopsin clades: XLR (3 genes), NaR (1 gene), XeR (15 genes), and CyHR (24 genes) and one novel clade (13 genes) (Table 1 and Fig. 1a and Supporting Information Fig. S1); the novel rhodopsin clade consisted entirely of rhodopsin genes from cyanobacterial genomes so we named the clade “cyanorhodopsin” (CyR) (Fig. 1b).

**Table 1:** Rhodopsin distribution and habitats in cyanobacterial lineage.

Subclade	Rhodopsin-possessing											All
	Rhodopsin						Habitat				Genome	Genome
	XLR	NaR	XeR	CyHR	CyR	Total	Marine	Freshwater	Saline	NA^*^		
A, C, F, G	0	0	0	0	0	0	0	0	0	0	0	56^†^
B	1	1	12	18	12	44	2	21	9^‡^	2	34	85
D	1	0	2	5	1	9	0	6	0	0	6	7
E	0	0	1	1	0	2	0	1	0	0	1	5
NA^*^	1	0	0	0	0	1	0	1	0	0	1	1
Total	3	1	15	24	13	56	2	29	9	2	42	154

^*^NA = “not available”.

^†^The genomes are included as follows: subclade A(13), C(36), F(4), and G(3).

^‡^Eight of the nine genomes are derived from the strains isolated at the same site, with little genetic variation.

Cyanobacterial nomenclature is based largely on morphological features; therefore, species names often do not reflect their lineage. Therefore, to examine the rhodopsin gene distribution we reconstructed a phylogenomic tree of the 154 cyanobacteria genomes by using conserved phylogenetic marker proteins (120 ubiquitous single-copy proteins). Seven subclades (A–G) were assigned to the phylogenomic tree according to a previous study^21^, together with information on source environment, morphology, presence or absence of the retinal biosynthetic gene *diox1* (carotenoid oxygenase), and genome size (Supporting Information Fig. S2). Almost all of the genomes were found to encode *diox1* (152/154), indicating that microbial strains with rhodopsins generally also have the ability to produce retinal. In addition, we found that the rhodopsin-possessing strains were not evenly distributed across the subclades (Supporting Information Fig. S2, Table 1 and Supporting Information Table S1): strains in subclades A, C (mainly marine cyanobacteria with relatively smaller genomes), F, and G did not possess any rhodopsin genes; in contrast, almost all the strains in subclade D did possess rhodopsin genes and subclade B contained all functional types of cyanobacterial rhodopsin detected in this study (Supporting Information Fig. S2 and Table 1). No morphological bias in rhodopsin distribution was observed (Supporting Information Fig. S2).

### Amino acid sequences and functions of the rhodopsins in the CyR clade

To examine the functions of the rhodopsins in the CyR clade, a motif sequence containing specific amino acid residues that are crucial for ion transport activity was examined. In BR, the motif corresponds to Asp85^BR^, Thr89^BR^, and Asp96^BR^ (DTD) in the third helix (helix C); the Asp85 and Asp96 residues work as proton acceptor and donor, respectively, and Thr89 forms a hydrogen bond with Asp85. Of the 13 rhodopsins in the CyR clade, the DTD motif was detected in 10, whereas the corresponding motif was Asp, Thr, Glu (DTE) in *Calothrix* sp. NIES-2098, *Nostoc* sp. RF31Y, and *Nostoc* sp. 106C (Supporting Information Fig. S3). All of the CyRs included Lys204^N2098R^ in the seventh helix (helix G), which is known to make a Schiff base linkage between the rhodopsin protein moiety and the retinal chromophore in other rhodopsins (Supporting Information Fig. S3). Also, an aspartic acid residue (Asp200^N2098R^) and two glutamic acid residues (Glu182^N2098R^ and Glu192^N2098R^), which are classified as a counterion stabilizing the Schiff base and a proton release group, respectively, were conserved in BR and all of the CyRs^22,23^. Based on this analysis, CyRs were expected to function as light-driven H^+^ pumps.

**Figure 2:**
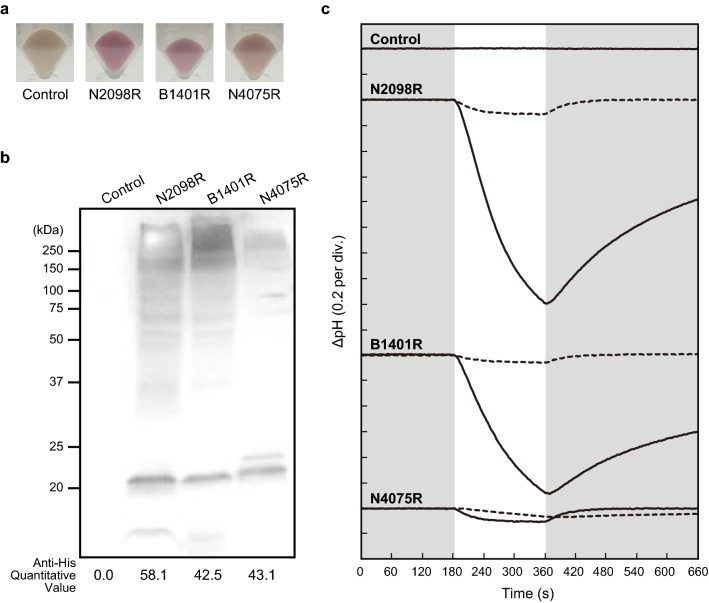
Light-induced changes of the pH of suspensions of *Escherichia coli* expressing a rhodopsin (N2098R, B1401R, and N4075R) from the novel CyR clade. (**a**) The pellet color of CyRs. (**b**) Detection of protein expression of CyRs by western blots using an anti-His-tag antibody. These proteins were expressed in *E. coli* cells with a His-tag at the C-terminal. The monomer-band of CyRs (around 22 kDa) were quantified using ImageJ software. (**c**) The changes in pH in the absence (solid line) and presence (broken line) of CCCP are shown. The numbers in parentheses are the pH units of *y*-axis divisions. All measurements were performed under the dark condition (gray shading) with illumination at 520 ± 10 nm for 3 min (white shading). ﻿*E. coli* cells containing the pET21a plasmid vector alone were simultaneously analyzed as a negative control.

Next, to further examine the ion-transporting activities of the CyRs, we heterologously expressed three synthesized rhodopsin genes—N2098R (BAY09002.1), B1401R (WP_074382570.1), and N4075R (GAX43141.1)—in *Escherichia coli* (see Fig. 1b). Rhodopsin-expressing *E. coli* cells showed more colors than control vector (pET21a) (Fig. 2a) and protein expressions were detected by western blots using anti-His-tag antibody (Fig. 2b). We examined the light-induced change in the pH of the cell suspension. A light-induced decrease in pH was observed in the suspensions of the cells expressing each of the rhodopsins (Fig. 2c, solid line), and this decrease was almost completely abolished in the presence of the protonophore carbonyl cyanide *m*-chlorophenylhydrazone (CCCP) (Fig. 2c, broken line). These results showed that N2098R and B1401R transport proton from the cytoplasmic side to the extracellular space. On the other hand, N4075R was thought to act as an outward proton pump, but its activity was smaller than that of the others. Therefore, the possibility of transporting other ions cannot be excluded.

### Spectroscopic characterization of N2098R

**Figure 3:**
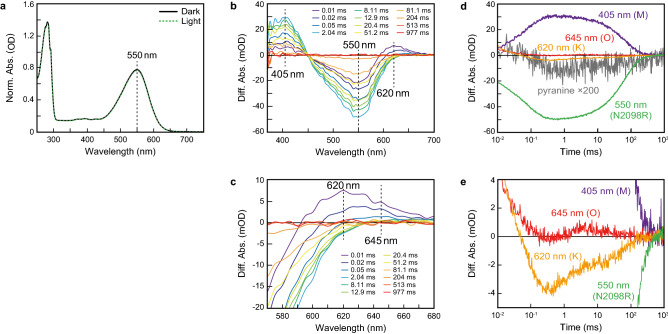
Absorption spectra and photocycle of N2098R. (**a**) UV–Vis spectra of N2098R with (green broken line) and without (black solid line) light illumination at 550 ± 10 nm for 10 min. (**b**) Flash-induced difference absorption spectra of N2098R over a spectral range of 370 to 700 nm and a time range of 0.01 to 977 ms. (**c**) Detail of flash-induced difference absorption spectra of N2098R over a spectral range of 570 to 680 nm and a time range of 0.01 to 977 ms. (**d**) Flash-induced kinetic data of N2098R at 405 nm (violet line), 550 nm (green line), 620 nm (orange line), and 645 nm (red line). The gray line represents the absorption changes of pyranine monitored at 450 nm. (**e**) Detail of flash-induced kinetic data of N2098R at 405 nm (violet line), 550 nm (green line), 620 nm (range line), and 645 nm (red line).

To characterize the photochemical properties of the CyRs, we focused on N2098R, a well-expressed, stable rhodopsin. After adaption of purified N2098R to the light or dark condition, the absorption maxima of both adapted samples of N2098R was located at 550 nm (Fig. 3a), which was similar to that of GR but not to that of BR or PR (Table 2)^6,16,24^.

**Table 2:** Photochemical properties of light-driven outward proton pump rhodopsins.

Opsin type	λmax (nm)	Retinal configuration	M-decay rate constant	p*K*_a_ value
N2098R	550	All-*trans* (predominant)	Approx. 0.016 ms^−1^	Below 2.0 (Asp74) Approx. 10.7 (Lys204)
BR	570^24^	All-*trans* (apprx. 50%)^27^ 13-*cis* (approx. 50%)^27^	Approx. 0.25 ms^−135^	Approx. 2.6 (Asp85)^29^ Approx. 13.3 (Lys216)^32^
PR (GPR)	520^6^	All-*trans* (predominant)^25^	Approx. 4 ms^−125^	Approx. 7.9 (Asp97)^30^ Approx. 11.3 (Lys227)^33^
PR (BPR)	490^42^	N.D.	N.D.	Approx. 6.2 and 7.9 (Asp97)^31^ N.D. (Lys227)
GR	544^16^	All-*trans* (predominant)^15,26^	Approx. 2.3 ms^−115^, Approx. 1 ms^−136^	Approx. 5.9 (Asp121)^16^ Approx. 9.2 (Lys257)^16^

Next, we examined the retinal configuration in N2098R by high-performance liquid chromatography. Both in light- and dark-adapted samples, the isomeric state of retinal was predominantly all-*trans* (Supporting Information Fig. S4 and Table 2), which was similar to the isomeric state of retinal in PR^25^ and GR^15,26^ but different from that in BR (Table 2)^27^.

Charged residues (e.g., Asp85^BR^ and Lys216^BR^ in BR) are essential for proton transportation by rhodopsins^28^. We therefore estimated the p*K*_a_ values of the charged residues in N2098R (i.e., Asp74^N2098R^ and Lys204^N2098R^) by pH titration and fitted the data using the Henderson–Hasselbalch equation assuming a single p*K*_a_; the p*K*_a_ values of Asp74^N2098R^ and Lys204^N2098R^ were estimated to be < 2.0 and 10.7, respectively (Supporting Information Fig. S5 and Table 2). The p*K*_a_ of Asp74^N2098R^ was much smaller than that reported for the equivalent amino acid in other microbial rhodopsins^16,29–31^, whereas the p*K*_a_ of Lys204^N2098R^ was comparable with that reported for the equivalent amino acid in other microbial rhodopsins (Table 2)^16,32,33^.

When microbial rhodopsins absorb visible light, the retinal chromophore is isomerized from the all-*trans* to the 13-*cis* form and the rhodopsin protein then forms various photointermediates before returning to its original state. These photochemical reactions, which occur within the picosecond-to-second time frame, are collectively referred to as the photocycle. In BR, several photointermediates (designated as intermediates K, L, M, N, and O) have been identified^34^. To examine the ion-transportation mechanism of N2098R, the photochemical reactions it undergoes were examined by flash-photolysis analysis. The flash-induced difference spectra of purified N2098R showed bleaching and recovery to the original state within 977 ms at a wavelength of around 550 nm (Fig. 3b), which coincided with the absorption maxima of N2098R (Fig. 3a). Two large positive peaks were observed at around 620 and 405 nm; the absorbance at around 620 nm appeared within 0.01 ms and the absorbance at around 405 nm appeared within 2.04 ms (Fig. 3b). In addition, one small positive peak was observed at around 645 nm; the absorbance at around 645 nm appeared with in 81.1 ms (Fig. 3c).

Next, the time courses of the changes of absorbance at wavelengths of 405, 550, 620, and 645 nm were examined (Fig. 3d and e). From the time and location of the absorption maxima, the increases and decreases at around 620, 405, and 645 nm were attributed to the formation and decay of the K-, M-, and O-intermediates, respectively. That is, when the K-intermediate was bleached, the M-intermediate began to form; then when the M-intermediate decayed, the O-intermediate began to form. After then, O-intermediate was bleached and the rhodopsin protein returned to its original state (550 nm). The photocycle was complete by about 300 ms. The M-decay rate constant at pH 7.0 was estimated to be 0.016 ms^−1^ by fitting a single exponential equation. This decay rate constant was much smaller than that reported for PR (4 ms^−1^)^25^, BR (0.25 ms^−1^)^35^, and GR (2.3 ms^−1^ and 1 ms^−1^)^15,36^ indicating that the M-intermediate of N2098R was much longer-lived than that of these other rhodopsins (Table 2). When the same experiment was repeated under various pH conditions, the M-decay rate was markedly increased under acidic conditions, suggesting that proton concentration mediates the rate of decay of the M-intermediate (Supporting Information Fig. S6).

To clarify the timing of proton uptake and release during the photocycle, we monitored the changes in absorption of N2098R in the presence or absence of pyranine, a pH-sensitive dye^37^. The pyranine signal in the difference spectrum decreased within 0.1 ms and then increased within 100 ms (Fig. 3d, gray line); these curves coincided well with the M-formation (K-decay) and M-decay (recovery of the original state). Because decreases and increases of the pyranine signal reflect the acidification and alkalization, respectively, of the bulk solution, this finding indicates that a proton is released from N2098R upon M-formation and is taken up from the bulk solution upon M-decay. The order of the timing is the same as that of BR^38^, but different from that of PR^25^.

### Structure of N2098R and comparison with those of BR, GR, and ASR

**Figure 4:**
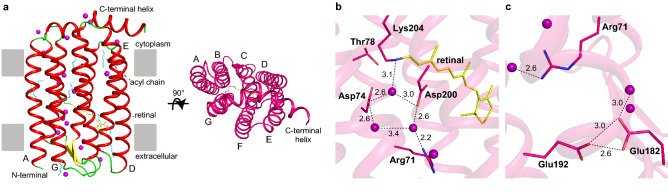
Structure of N2098R. The retinal molecule and acyl chains are shown as yellow and cyan stick models, respectively. Water molecules are shown as purple spheres. (**a**) Crystal structure of N2098R, viewed parallel to the membrane (left panel) and from the cytoplasmic side (right panel). (**b**) Structure of the retinal binding and water cluster region. (**c**) Structure of the extracellular portion of the proton translocation pathway; the region near the putative proton efflux region is shown. Numbers indicate the distance (Å) between two atoms connected by dashed lines.

The crystal structures of cell-free-synthesized N2098R and N4075R were determined at 2.65 Å and 1.9 Å resolution, respectively (Supporting Information Table S2). Since the two structures were almost identical, only the structure of N2098R is described (Fig. 4 and Supporting Information Fig. S7a–c). The N2098R protein crystallized into space group *C222*_*1*_, with a N2098R trimer per asymmetric unit. The N2098R monomer is composed of seven transmembrane helices (helices A–G) and a long C-terminal helix (Fig. 4a). The long C-terminal helix is bent at the end of helix G, Gly215^N2098R^, and covers the cytoplasmic surface of helix C–F (Fig. 4a, right panel). However, this bend of the C-terminal helix could be due to crystal packing, so it is unclear whether the C-terminal helix of N2098R is bent under physiological conditions.

The overall structure of N2098R is similar to that of BR (PDB code 1C3W)^39^ (root-mean-square deviation [RMSD] 0.995 Å), except for the presence of the long C-terminal helix in N2098R (Supporting Information Fig. S7d and Supporting Information Table S3). The structure around the retinal in N2098R and BR is shown in Fig. 4b and Supporting Information S7e; in both rhodopsins, the retinal is unbent. A pentagonal cluster consisting of three water molecules adjacent to the Schiff base of Lys204, Asp74, and Asp200 plays an important role in proton transport (Fig. 4b, broken lines). This water cluster near the retinal is similar to that next to the Schiff base of BR (Lys216^BR^, Asp85^BR^, and Asp212^BR^) (Supporting Information Fig. S7e). In addition, the location of the putative proton release group comprising Glu182 and Glu192 (corresponding to Glu194^BR^ and Glu204^BR^ in BR) is also very similar (Supporting Information Fig. S7f.).

The structure of N2098R was also compared with the cyanobacterial rhodopsin GR (PDB code 6NWD)^40^ and ASR (PDB code 1XIO)^41^, which are included in the XLR and XeR rhodopsin clades, respectively. The overall structure of N2098R is similar to that of GR (RMSD 1.91 Å) and ASR (RMSD 1.40 Å) (Supporting Information Fig. S7g and j and Supporting Information Table S3), but the structural position of the water molecules and amino acids around the retinal and the putative exit region of the proton are quite different (Supporting Information Fig. S7h, I, k, and l). These structural differences also suggest that CyR including N2098R is a “unique” proton-pumping rhodopsin, unlike known cyanobacterial rhodopsin such as GR and ASR.

To examine the amino acid residues crucial for the proton-transport activity of N2098R, mutants at the putative proton acceptor Asp74^N2098R^, proton donor Glu85^N2098R^, or counterion Asp200^N2098R^ were examined. Replacement of these amino acids prevented light-mediated change of the pH of the cell suspensions despite expressed as well as wild type in *E. coli* cells, indicating that these residues are essential for proton transport (Supporting Information Fig. S8); this finding provides further evidence that N2098R functions as a light-driven outward proton pump. In addition, deletion-mutation analysis of the C-terminus (1–125) indicated that the helix is important for structural stabilization (Supporting Information Fig. S8).

## Discussion

Since the discovery of PR in 2000, many genomics-based studies have shown the widespread distribution of rhodopsin genes in prokaryotes, which has greatly expanded our understanding of light-energy utilization mechanisms in microbial ecosystems. In the present study, we examined the rhodopsin gene distribution in the cyanobacteria lineage. A homology search of 154 cyanobacterial genomes with associated source environment and strain morphological information detected 56 rhodopsin genes (Table 1) in 42 genomes and revealed that rhodopsin genes are widespread among cyanobacterial strains, particularly in freshwater dwellers. Construction of a phylogenomic tree allowed us to clarify the habitat-biased distribution of rhodopsin genes (Table 1 and Supporting Information Fig. S2), suggesting that each cyanobacterial subclade has developed a different light adaptation strategy. For example, subclade C comprised strains that did not possess rhodopsin genes and were mainly marine cyanobacteria (i.e., *Prochlorococcus* and *Synechococcus*) that inhabit a oligotrophic environment; thus, although the availability of nutrients such as nitrogen and phosphorus limits photosynthesis, these organisms are thought to have adapted to such environments not by evolving an auxiliary photosystem, but by minimizing the resources needed for survival through extensive genome streamlining. On the other hand, in freshwater, which usually has a stable light supply and a variable but relatively abundant supply of nutrients, rhodopsin-mediated phototrophy may have some survival advantages.

The identified cyanobacterial rhodopsins were classified into five clades, including a novel clade (CyR), and these distributions were polyphyletic (Fig. 1a and Supporting Information Fig. S1), indicating that rhodopsin genes have been gained or lost more than once during evolution. In particular, the strains in clades NaR (1 gene detected in this study) and XLR (3 genes detected), which are unlikely to have diverged during speciation, may have obtained their rhodopsin genes via horizontal gene transfer. On the other hand, rhodopsins XeR, CyHR, and CyR are likely to have evolved within the cyanobacterial lineage because of their clustered characteristics, suggesting that these rhodopsins may have been optimized for cyanobacterial cells. Notably, almost all of the cyanobacteria genomes examined carried the *diox1* gene, which encodes a protein that converts apo-carotenoids to all-*trans* retinal. Whether retinal has a role other than as the chromophore in rhodopsin in vivo remains unclear, but the widespread distribution of *diox1*, even in strains lacking rhodopsin, means that almost all cyanobacteria are capable, genetically at least, of constructing a rhodopsin-mediated photosystem simply by acquiring the rhodopsin gene. This finding also supports the acquisition of rhodopsin genes within the cyanobacterial lineage via horizontal gene transfer.

Heterologous rhodopsin gene expression analysis showed that the rhodopsins in the CyR clade function as light-driven outward H^+^ pumps. Although H^+^-pumping rhodopsins have been found in cyanobacteria (e.g., GR in clade XLR)^14–16^, H^+^ pumps unique to cyanobacteria were previously unknown. Therefore, we conducted spectroscopic and structural analyses of N2098R to evaluate the possibility that the rhodopsins in the CyR clade are fine-tuned specifically for freshwater photoautotrophs. First, N2098R had an absorption peak at 550 nm, much like the GR, but not the BR of halophilic archaea or the PR of marine bacteria (Table 2). Chlorophyll cannot directly use green light at around 550 nm, making green light ideal for CyRs. PR and BR use light with a wavelength shorter than 550 nm and longer than 550 nm, respectively, presumably because they have evolved to use the light specific to the environment their expressing strains inhabit^24,31,42^. pH titration experiments showed that the p*K*_a_ value of the putative proton acceptor Asp74^N2098R^ (< 2.0) was much lower than that of the corresponding amino acid in BR (Asp85; 2.6) and PR (Asp97; 7.9) (Table 2). This low value indicated that Asp74^N2098R^ is deprotonated and can accept protons over a wide pH range, which may be desirable in freshwater environments because the pH of terrestrial water tends to fluctuate between neutral or weakly acidic, whereas that of seawater tends to remain slightly alkaline. Furthermore, elucidation of the photocycle of N2098R revealed that the M-decay rate, an index of the protonation of the Schiff base, of N2098R was much smaller than that of PR under neutral or weakly alkaline conditions, but was increased under acidic conditions (Table 2 and Supporting Information Fig. S6b). Together, the values of p*K*_a_ and M-decay rate indicate that N2098R is particularly suitable for weakly acidic conditions. A structural comparison revealed a high conformational similarity between N2098R and BR, and a mutant analysis confirmed that the putative proton donor and acceptor in N2098R both worked as expected. These results indicate that N2098R differs from known H^+^-pumping rhodopsins in many aspects, including maximum absorption wavelength, p*K*_a_, and M-decay rate, even though its structure is almost identical to that of BR, suggesting that these characteristics may have been uniquely tuned to the cyanobacterial habitat via slight changes in the structure of the side chains.

Many cyanobacteria are known to possess elaborate antenna complexes, such as phycobilisome, which efficiently capture green light that cannot be absorbed by chlorophyll^2^. Nevertheless, the CyRs in the present study and the previously reported CyHRs^17,18^ have also been shown to absorb green light at around 550 nm. This suggests that there is an environment or physiological state in which rhodopsin absorbs green light more effectively than do the other antenna complexes. Although the expression pattern and intracellular localization of CyRs must be examined to elucidate their cell biology, CyRs, like other light-driven H^+^ pumps, appear to be involved in light-energy acquisition in connection with ATP synthase. In fact, it has recently been reported that the growth rate of a *Synechocystis* sp. PCC 6803 mutant lacking PSI and expressing heterologous PR is improved under green light illumination, demonstrating that a rhodopsin-mediated photosystem acts as a light-energy acquisition device in cyanobacterial cells^43^.

A series of photosynthetic reactions initiated by the excitation of chlorophyll was thought to be the only photoenergy-capturing mechanism within cyanobacterial cells. Through the present large-scale genomic and heterologous expression analyses, we examined the distribution and function of rhodopsin, an auxiliary light-energy capture device, in the cyanobacterial lineage. Our surveys revealed not only that rhodopsin genes are present in cyanobacteria, predominantly in freshwater cyanobacteria, but also that CyRs functions as a light-driven H^+^ pump. Spectroscopic observation suggested that the features of these rhodopsins are such that the proteins do not interfere with chlorophyll absorption and are specifically adapted for freshwater environments. The biased rhodopsin distribution indicated that cyanobacteria have invested in the acquisition and development of chlorophyll-independent light-harvesting devices. These findings open up new avenues of research with respect to the light-utilization mechanisms within bacterial cells and provide a better understanding of the evolutionary history of cyanobacterial photosystems.

## Materials and methods

Detailed materials and methods are provided in Supplementary information.

A sequence homology search for rhodopsins was conducted using 154 cyanobacterial genomes, and a phylogenetic tree of rhodopsins was constructed with RAxML (v.8.2.11)^44^ using 100 rapid bootstrap searches.

Codon-optimized DNA fragments for *E. coli* encoding the novel rhodopsins were chemically synthesized by Eurofins Genomics (Tokyo, Japan) and inserted into the pET21a (+) plasmid vector (Novagen, Darmstadt, Germany). Protein expression of CyRs in *E. coli* cells were detected by western blots using an anti-His-tag antibody. The ion-transport activities of the novel rhodopsins were examined by monitoring the light-induced changes of pH in suspensions of CyR-expressing *E. coli* in 100 mM NaCl.

CyR-expressing *E. coli* were solubilized with 1.0% (w/v) *n*-dodecyl-β-d-maltoside (DDM, Dojindo Lab., Kumamoto, Japan) and purified with a HisTrap FF Ni^2+^-NTA affinity chromatography column (GE Healthcare, Amersham Place, England). Retinal isomer composition was determined by high-performance liquid chromatography as previously described^45^.

For p*K*_a_ determination, the pH of N2098R was adjusted to pH 1.00–11.52 by the addition of a small amount of 1 N HCl or NaOH and the absorption spectrum at 250–750 nm was measured over the pH range. p*K*_a_ was then estimated by fitting the data to the Henderson–Hasselbalch equation.

For flash-photolysis analysis, time-resolved absorption spectra from 370 to 700 nm at 5 nm intervals were measured by using a computer-controlled flash-photolysis system, and pyranine was used to observe proton uptake and release during the photocycle.

N2098R and N4075R for crystallization was synthesized by using an *E. coli* cell-free protein synthesis system and the protein was crystallized by the *in meso* method. Diffraction data of N2098R and N4075R were collected at BL32XU of the SPring-8 synchrotron (Hyogo, Japan) by using the multiple small-wedge scheme implemented in the ZOO system^46,47^, and data processing was performed using KAMO^48^. The structure was solved by molecular replacement using the Phaser program^49^ in the Phenix suite^50^.

## Supplementary information

Supplementary file1

## Data Availability

The coordinates and structure factor for N2098R and N4075R have been deposited in the Protein Data Bank, www.wwpdb.org (PDB ID code 6LM0 and 6LM1).
