# Supplementary material for: A unique clade of light-driven proton-pumping rhodopsins evolved in the cyanobacterial lineage
Source: Sci Rep. 2020 Oct 7;10:16752. doi: 10.1038/s41598-020-73606-y (PMC7541481; doi:10.1038/s41598-020-73606-y)
Supplement: Supplementary file 1 — Supplementary file1 [file 41598_2020_73606_MOESM1_ESM.pdf]

Supplementary Information for

A unique clade of light-driven proton-pumping rhodopsins evolved in the cyanobacterial lineage

Masumi Hasegawa, Toshiaki Hosaka, Keiichi Kojima, Yosuke Nishimura, Yu Nakajima, Tomomi Kimura-Someya, Mikako Shirouzu, Yuki Sudo, Susumu Yoshizawa*

*Corresponding Author: yoshizawa@aori.u-tokyo.ac.jp

This file includes

Supplementary Materials and Methods p. 2–7
Supplementary Results p. 8–11
Supplementary Figures 1–8 p. 12–22
Supplementary Tables 1–3 p. 23–25
Supplementary References p. 26–28

Supplementary Materials and Methods

Genomic and phylogenetic analysis

A rhodopsin phylogenetic tree was constructed as follows. Genome assembly data and rhodopsin sequences were collected from the National Center for Biotechnology Information and the U.S. Department of Energy Joint Genome Institute Integrated Microbial Genomes database. The quality of the genomes was assessed using CheckM (v.1.0.11) [1]. A sequence homology search for rhodopsins in the set of cyanobacterial genomes was performed by using hmmsearch (included in the HMMER package, v.3.1b2) with the e-value threshold set to 1e-10 [2]. The profile HMMs used for the queries were created as follows. The sequences were aligned using T-COFFEE (http://tcoffee.crg.cat/) in accurate mode. The phylogenetic tree was inferred using RAxML (v.8.2.11) [3] with the PROTGAMMAVTF model using 100 rapid bootstrap searches. Model selection was performed with the ProteinModelSelection.pl script in the RAxML package.

A cyanobacterial genome phylogenetic tree was constructed as follows. A multiple sequence alignment of conserved phylogenetic marker proteins (ubiquitous single-copy proteins) were computed using the 'classify_wf' workflow of GTDB-Tk (v.0.1.1) [4]. The genome phylogenetic tree was computed with the 'PROTGAMMALG' model of RAxML using 100 rapid bootstrap searches. This model was selected as the best model by the ProteinModelSelection.pl helper script in the RAxML package.

The phylogenetic trees were visualized with Interactive Tree Of Life (v.3) [5]. Habitat and morphology information was collected manually.

Construction of plasmids

Codon-optimized DNA fragments for *Escherichia coli* encoding the novel rhodopsins were chemically synthesized by Eurofins Genomics (Tokyo, Japan) and inserted into the pET21a (+) plasmid vector (Novagen, Darmstadt, Germany) using the NdeI and XhoI restriction enzyme sites. This cloning strategy resulted in encoding hexahistidines at the C-terminus.

Site-directed mutations (D74N, E85Q, and D200N) and C-terminus-deletion mutations (amino acids 1–215 or 1–224) were introduced separately into the N2098R gene subcloned into the pET21a (+) plasmid vector (Novagen).

Functional analyses of representative CyRs and N2098R mutants

In this study, we synthesized rhodopsin genes—N2098R (BAY09002.1), B1401R (WP_074382570.1), N4075R (GAX43141.1), and Cyanobacteria bacterium QS_4_48_99 (PSO81870.1) — and used them for heterologous expression experiments. These rhodopsin genes were inserted into the pET21a (+) plasmid vector (Novagen, Darmstadt, Germany). The plasmids and N2098R mutants plasmids were transformed into *E. coli* strain C41 (DE3) (Lucigen, Middleton, WI, USA) cells and were incubated at 37°C on an LB medium agar plate containing 100 μg mL^{-1} ampicillin. Before protein expression, transformants were grown at 37°C in 100 mL of 2× YT medium with 100 μg mL^{-1} ampicillin until the absorbance at 660 nm had reached 0.4–0.9. Protein expressions were then induced at 37°C for 3–4 h by adding 0.1 mM isopropyl β-D-1-thiogalactopyranoside and 10 μM all-*trans* retinal (Sigma-Aldrich, St. Louis, MO, USA). However, the rhodopsin gens (PSO81870.1) was not expressed in *E. coli* and therefore was not used in subsequent analyses. The rhodopsin-expressing cells were collected by centrifugation (4400 × g for 3 min), washed three times in 100 mM NaCl, and resuspended in 6 mL 100 mM NaCl for measurements. The cell suspension was placed in the dark until the pH of the sample had stabilized and was then illuminated by using a 300-W xenon lamp (MAX-303, Asahi spectra, Co., Ltd., Japan) with a green band-pass filter (520 ± 10 nm; MX0520, Asahi spectra, Co., Ltd., Japan) for 3 min. Light-induced pH changes were monitored with a pH electrode (LAQUA F-72 pH meter, HORIBA, Ltd., Kyoto, Japan) under the presence or absence of a protonophore, 30 μM carbonyl cyanide *m*-chlorophenylhydrazone (Sigma-Aldrich), to confirm that the changes in pH were caused by proton transport. All measurements were performed at 4°C.

SDS-PAGE and western blot analysis

For detection of protein expression, sodium dodecyl sulfate-polyacrylamide gel electrophoresis (SDS-PAGE) and western blot analysis were carried out. Rhodopsin-expression *E. coli* C41 (DE3) cells were resuspended in 100 mM NaCl and these total proteins were extracted using EzBactYeast Crusher (ATTO Co., Tokyo, Japan) according to standard method. These extract samples were optimized these densities using DC™ Protein Assay Kit I (Bio-Rad Lab., Inc., Hercules, CA, USA). These samples mixed with an equal volume of 2× Laemmli Sample Buffer (Bio-Rad) containing 2-mercaptoethanol (final concentration = 355 mM) were denatured by

heating (95°C, 5 min), and the supernatants collected by centrifugation (20,400 × *g* for 10 min) were subjected to 12%-acrylamide SDS-PAGE. Immunoblotting analysis was then performed using an Anti-His-tag mAb-HRP-DirecT (Medical & Biological Laboratories Co., Ltd., Nagoya, Japan) by the standard method. Antibody binding was detected by chemiluminescence using Clarity™ Western ECL Substrate (Bio-Rad) and visualized by Omega Lum G (Aplegen, San Francisco, CA, USA). These were quantified using ImageJ software (http://rsb.info.nih.gov/ij/).

Protein purification from E. coli and spectroscopic analysis of N2098R

For protein purification, N2098R-expressing *E. coli* C41 (DE3) cells were resuspended in 7 mL buffer containing 50 mM Tris–HCl (pH 8.0) and 500 mM NaCl. The cells were then disrupted by sonication (UD-200, TOMY Seiko Co., Ltd., Tokyo, Japan) on ice-cold water. Crude membranes were obtained by ultracentrifugation at 4°C (106,800 × *g* for 30 min; Optima XPN-90 Ultracentrifuge with SW 32Ti rotor, Beckman Coulter, USA) and solubilized with 1.0% (w/v) *n*-dodecyl-β-D-maltoside (DDM; Dojindo Lab., Kumamoto, Japan). The solubilized N2098R was collected as the supernatant after ultracentrifugation at 4°C (106,800 × *g* for 30 min) and purified with a HisTrap FF Ni^{2+}-NTA affinity chromatography column (GE Healthcare, Amersham Place, England) at room temperature (approx. 25°C). The purified sample was concentrated, and its buffer exchanged to a new buffer (10 mM Tris–HCl (pH 7.0), 50 mM NaCl, 0.05% DDM) adjusted to 0.5 optical density mL^{-1} at 550 nm by using an Amicon Ultra Filter (10,000 M_w cut-off; Millipore, Bedford, MA, USA) and centrifugation at 4°C.

UV–Vis spectra were measured using a UV-1800 spectrophotometer (Shimadzu, Kyoto, Japan) at room temperature (approx. 25°C).

Retinal isomer composition was determined by using high-performance liquid chromatography [6,7]. Retinal in the sample was extracted with hexane as retinal oxime after denaturation by methanol (final concentration = 66.7 % (v/v)) with hydroxylamine (final concentration = 100 mM). Before analysis, each sample was kept in the dark for more than seven days. For the light adaptation, the sample was illuminated at 550 ± 10 nm light for 10 min. The light power was adjusted to approximately 10 mW cm^{-2} by using an optical power meter (#3664, Hioki, Nagano, Japan). The molar composition of each retinal isomer was calculated from the areas of the peaks of the high-performance liquid chromatography chromatograms monitored

at 360 nm. The previously reported absorption coefficients of retinal isomers (51,600, 54,900, 52,100, and 49,000 cm^{-1} M^{-1} for all-*trans* 15-anti, all-*trans* 15-syn, 13-*cis* 15-anti, and 13-*cis* 15-syn retinal oximes, respectively) were used in the present calculation [7–10]. All measurements were conducted at room temperature (approx. 25°C) under red light (>600 nm).

pKa determination of N2098R

For the pK_a determination, purified N2098R was suspended in seven-mix buffer (Tris, citric acid, MES, HEPES, MOPS, CHES, and CAPS; 10 mM each) containing 50 mM NaCl and 0.05% DDM; this buffer has the same buffer capacity over a wide pH range. The initial pH of the buffer was approximately 6.8. Then, the pH was adjusted to the desired value (pH 1.00–11.52) by adding a small amount of 1N HCl or NaOH and the absorption spectrum (250–750 nm) was measured for samples under various pH conditions. All measurements were conducted at room temperature (approx. 25°C) under room light. Absorption difference (ΔAbs) at specific wavelengths was plotted against pH. Then, the acid dissociation constants (pK_a) were estimated by fitting the data to the Henderson–Hasselbalch equation as follows:

$$\Delta\mathrm{Abs} = \frac{v}{1+10^{(\mathrm{pH}-\mathrm{p}K\mathrm{a})}} + w$$

where v represents the amplitudes of the change of absorption differences and w is an offset. After the experiment, the reversibility of the pH-dependent spectral changes of the samples was checked to confirm that the protein was not denatured during the experiment.

Time-resolved transient absorption spectroscopy of N2098R

For the flash-photolysis experiment, the purified N2098R was concentrated, and its buffer exchanged to a new buffer (10 mM Tris-HCl (pH 7.0), 50 mM NaCl, 0.05% DDM) adjusted to 0.5 optical density mL^{-1} at 550 nm by using an Amicon Ultra Filter (10,000 M_w cut-off; Millipore, Bedford, MA, USA) and centrifugation at 4°C. Time-resolved absorption spectra from 370 to 700 nm at 5 nm intervals were measured by using a computer-controlled flash-photolysis system equipped with a Nd:YAG laser (Surelite I-10; Continuum, San Jose, CA) as an actinic light source and a multichannel detector (Hamamatsu Photonics K. K., Shizuoka, Japan). The wavelength of the actinic pulse light was tuned at 555 nm using an optical parametric oscillator (Surelite

OPO plus; Continuum). The other parameters were set as previously described [11]. Data taken before the flash were used as the baseline. At each wavelength, 20 traces were averaged to improve the signal-to-noise ratio. The temperature of the sample was kept at 25°C using a thermostat.

To observe proton uptake and release during the photocycle, pyranine (final concentration = 100 μM; Tokyo Chemical Industry Co., Ltd., Tokyo, Japan), which has often been used to monitor light-induced pH changes in various rhodopsins (e.g., S. Inoue et al. 2018 [12]), was used as a pH indicator. The pH changes of the bulk environment were monitored as the absorption changes of pyranine at 450 nm, which were determined as the difference of absorption changes between the samples with and without pyranine. A total of 2400 traces were averaged to improve the signal-to-noise ratio. The temperature of the sample was kept at 25°C using a thermostat.

N2098R and N4075R protein crystallization

DNA fragments containing the N2098R and N4075R genes were amplified by polymerase chain reaction and sub-cloned into the expression vector pCR2.1–TOPO with a modified histidine tag for nickel resin and the cleavage site for tobacco etch virus (TEV) protease at the N-terminus [13].

The N2098R and N4075R proteins used for crystallization were synthesized by using an *E. coli* cell-free protein synthesis system according to previously reported protocols used for NM-R3 production [13–16]. After cell-free synthesis reaction and solubilization with DDM, both proteins were affinity purified on Ni-NTA Superflow resin (Qiagen). The His-affinity tag was cleaved off with TEV protease, the cleaved tag and the His-tagged TEV protease were removed by passage though Ni-NTA Superflow resin, and the N2098R and N4075R proteins were recovered from the flow-through fraction. The protein solution was concentrated and applied to a Superdex 200 10/300 column (GE Healthcare). The peak fractions containing the protein were pooled and concentrated again.

N2098R (81.3 mg/mL) and N4075R (75.9 mg/mL) proteins were crystallized by the *in meso* method. The purified protein solution and monoolein (40:60 w/w) were homogenized and the mixture was placed on a glass well plate using a micro-dispenser as previously described [13,17]. Crystals of N2098R were grown at 20°C in 0.1 M Na-acetate (pH 5.0–5.5), 200–250 mM Na-chloride, and 39%–40% polyethylene glycol 500. Crystals of N4075R were also grown at 20°C in 0.1 M MES (pH 6.1–6.3),

450 mM magnesium nitrate, and 27% polyethylene glycol 500.

Data collection, structure determination, and refinement

Diffraction data for N2098R were collected at the BL32XU beamline of the SPring-8 synchrotron by using the multiple small-wedge scheme implemented in the ZOO system [18,19]. A total of 317 small-wedge datasets were collected at 10° using a 5 × 5-μm beam. Data processing was performed using the KAMO program [20] and data from 214 isomorphous small crystals were merged together to generate the final 2.65 Å dataset [21–23]. Diffraction data for one crystal of N4075R were also collected at the BL32XU beamline. The structures were solved by molecular replacement using the Phaser program [24] in the Phenix suite [25]. The search model was the BR trimer [26] and the structure was manually rebuilt with the Coot program [27]. Data collection and refinement statistics are presented in Table S2.

Supplementary Results

pK_a estimation

Charged residues (e.g., $Asp85^{BR}$ and $Lys216^{BR}$ in BR) play essential roles in proton transportation [28]; therefore, we determined the pK_a values of the charged residues in N2098R ($Asp74^{N2098R}$ and $Lys204^{N2098R}$) by pH titration analysis.

$Asp74^{N2098R}$ is a putative primary proton acceptor, and so is usually deprotonated [29]; therefore, this pH titration analysis was performed under acid conditions (pH 6.81–1.00). A spectral red-shift was observed as the pH was lowered (Fig. S5a). Difference absorption spectra were calculated by subtracting the values obtained at pH 6.81, and two large peaks were observed at 514.5 and 597 nm (Fig. S5b). The absorption changes at 514.5 and 597 nm were plotted against pH and plots below pH 3.0 were fitted to the Henderson–Hasselbalch equation on the assumption of a single pK_a value (Fig. S5c). The pK_a value of $Asp74^{N2098R}$ was estimated to be 0.4, which is lower than that of the corresponding amino acid in BR (Asp85), PR (Asp97), and GR (Asp121) (Table 2) [30,31]. However, since the estimated pK_a was not sufficiently reliable due to the lack of point measured below pH 1.0, we concluded that the pK_a value of $Asp74^{N2098R}$ is less than 2 (Table 2).

$Lys204^{N2098R}$ is a putative binding site for retinal, which is covalently bound as a Schiff base and usually protonated [29]; therefore, this pH titration analysis was performed under alkaline conditions (pH 6.82–11.52). A large spectral blue-shift was observed as the pH was increased (Fig. S5d). Difference absorption spectra were calculated by subtracting the values obtained at pH 6.82, and two large peaks were observed at 363 and 550 nm (Fig. S5e). The absorption changes at 363 and 550 nm were plotted against pH and the data were fitted to the Henderson–Hasselbalch equation on the assumption of a single pK_a value (Fig. S5f). The pK_a value of $Lys204^{N2098R}$ was estimated to be 10.7, which is lower than that of BR ($Lys216^{BR}$) and PR ($Lys227^{PR}$), but higher than that of GR ($Lys257^{GR}$) (Table 2) [32–34].

Photocycle of N2098R

Proton uptake and release by N2098R during the photocycle were detected as the time course of the absorbance changes at 450 nm by using pyranine, a pH-sensitive dye. Pyranine works as a pH indicator, and the absorbance of pyranine at 450 nm was bleached under acidic conditions [35]. As a result, the absorbance at 450 nm (M-intermediate) was distinctly bleached and subsequently recovered as compared to the

signal of only rhodopsin or pyranine (Figs. 3d and S6c). This signal-to-noise ratio of the pyranine signal is comparable with our previous studies which could discuss the proton uptake and release processes of proton pumping rhodopsins [12,36]. Additionally, no detectable signal of pyranine was observed in the absence of the rhodopsin and in the presence of only the opsin, indicating that the pyranine signal in the presence of rhodopsin really significantly reflect the pH change of the bulk solution. These absorbance changes were observed coinstantaneous with that at 405 nm.

From the absorption maximum and the time period of the photocycle, we considered the absorbance changes at 620, 405, and 645 nm, observed by flash-photolysis analysis, to represent the K-, M-, and O-intermediates, respectively (Figs. 3b–e). In addition, the experiments using pyranine revealed that N2098R first releases a proton and then takes up a proton in its photocycle, simultaneous with the creation and bleaching of the M-intermediate (Fig. 3d). This manner of proton transport is identical to that of BR (archaeal proton pump) [37], but differs from that of PR (eubacterial proton pump) [38], and is considered to arise from the conservation of amino acids Glu182 and Glu192 in N2098R (Glu194 and Glu204 in BR), which are the proton release group in BR (Fig. S3). Thus, the N2098R photocycle proceeds as follows: (1) isomerization of retinal from all-*trans* to 13-*cis* induced by green light (550 nm); (2) creation and decay of K-intermediate; (3) creation of M-intermediate and release of a proton; (4) decay of M-intermediate and uptake of a proton; (5) creation and decay of O-intermediate; (6) recovery to the original state.

The proton acceptor $Asp74^{N2098R}$ has an exceptionally low estimated pK_a value (<2.0) (Fig. S5c), indicating that $Asp74^{N2098R}$ is deprotonated under neutral pH conditions. In addition, the low pK_a value of the Schiff base $Lys204^{N2098R}$ indicates that the retinal Schiff base can easily release a proton under neutral conditions (Fig. S5f). These photochemical features are very similar to those of BR, indicating that the rapid photocycle of N2098R and differences in pK_a values for N2098R from other proton-pumping rhodopsins are a result of slight structural changes compared with N2098R. Since the rate of decay of the M-intermediate at various pH showed no linear correlation with pH value below pH 5 (Fig. S6), it is likely that the proton does not come from the environment directly in this pH range. The several acidic and alkaline amino acid residues, such as Glu38, Glu85, and Asp104 in N2098R, located in the intracellular side to the Schiff base are candidates as proton donor residues. Among them, we propose that $Glu85^{N2098R}$ functions as a proton donor in N2098R

because the corresponding residue functions as a proton donor in various proton pumping rhodopsins, such as Asp96 in BR and Glu132 in GR (Fig. S3). On the other hand, the M-decay rates monotonically decreased above pH 5, which suggests that Glu85^{N2098R} is deprotonated and does not function as a proton donor in this pH range. Thus, the pK_a of Glu85^{N2098R} seems to be around 5 which is significantly lower than that of the proton donor of BR (Asp96^{BR}).

Structural characterization of N2098R

When we examined the overall structure of N2098R, we found that its structure resembled that of BR, and that the predicted functions of the essential amino acids in N2098R, as determined by comparison of amino acid sequences and crystal structure with BR, were supported by mutation analyses. N2098R covalently binds all-*trans* retinal in the dark by Schiff base linkage via Lys204^{N2098R}. This linkage is stabilized by deprotonated Asp74^{N2098R} and Asp200^{N2098R} as a counterion. Thus, color changes were observed for the D74N and D200N, and these absorption maxima were located at 590 nm and 560 nm, respectively (Figs. S8d and e). These red-shifts compared to WT (λ_{max} = 550 nm) were caused by protonation of these counterions. In addition, considering the range of red-shift, we think that D74 is a primary counterion and D200 is a secondary counterion. In addition, the absorption maxima of D74N was not red-shifted under the low pH (Fig. S8e). This result suggests that the red-shifts of Asp74^{N2098R} under the low pH (see Fig. S5a) was caused by protonation of D74. On the other hand, the absorption maxima of D74N were blue-shifted under the low pH. We think it was caused by protonation of secondary counterion (Asp200^{N2098R}) as reported in BR [39,40].

One major difference between N2098R and BR is that the hydrogen-bonding counterpart of the putative proton donor residue, Glu85^{N2098R} (Asp96^{BR}) in the cytoplasmic region, is Ser39^{N2098R} instead of Thr46^{BR} (Fig. S7e). In addition, there is a water molecule between the side chain of Glu85^{N2098R} (2.4 Å distance) and the side chain of Ser39^{N2098R} (2.6 Å distance). The side chain of Glu85^{N2098R} is stabilized via hydrogen bonds with the side chain of Ser39^{N2098R} and a water molecule.

Leu49^{N2098R} is located near the primary proton acceptor Asp74^{N2098R} in N2098R, and this feature is different from the structure of BR and PR (Figs. S7m, n, and o). Especially, in the case of PR, it is known that His75^{PR} forms a H-bond with the primary proton acceptor Asp97^{PR}, which explains its unusually high pK_a [41], and

these residues correspond to His57^{PR} and Asp79^{PR} in Fig. S7o. The low pK_a value of Asp74^{N2098R} is likely due to the absence of a hydrogen-bond between His75^{PR} and Asp97^{PR}, as in BR. In addition, Arg71^{N2098R} and Ser197^{N2098R} form a network of water molecules. It may also contribute to the lower pK_a of Asp74^{N2098R}, but further verification is required. Moreover, the monotonic decrease in M-decay (rate) above pH 5 suggested that the pK_a of the proton donor (Glu85^{N2098R}) is around 5 and deprotonated at neutral pH (Fig. S6b). Comparing the structure of N2098R and BR, a water molecule was closer to the proton donor in N2098R (Glu85) that in BR (Asp96) (Figs. S7a, b, and p). This interaction between the water molecule and the proton donor is thought to be responsible for the lower pK_a of Glu85^{N2098R}.

Furthermore, we found that N2098R has a long C-terminal extended helix, which is not conserved in other microbial rhodopsins, including BR and PR, but is similar to cytoplasmic helix eight of animal rhodopsins. In animal rhodopsins, cytoplasmic helix eight is anchored to the cell membrane by two Cys residues that are important for the interaction of light-activated rhodopsin with the retinal G-protein transducin [42,43], which acts as a membrane-dependent conformational switch [44,45]. Deletion-mutation analysis of the C-terminus suggested that the long C-terminal helix may support the proton-pumping activity and/or stability of the N2098R structure (Fig. S8c). However, the two Cys residues are not conserved in the C-terminal helix region of N2098R; thus, another mechanism is thought to be involved in stabilization of the structure.

Supplementary Figures

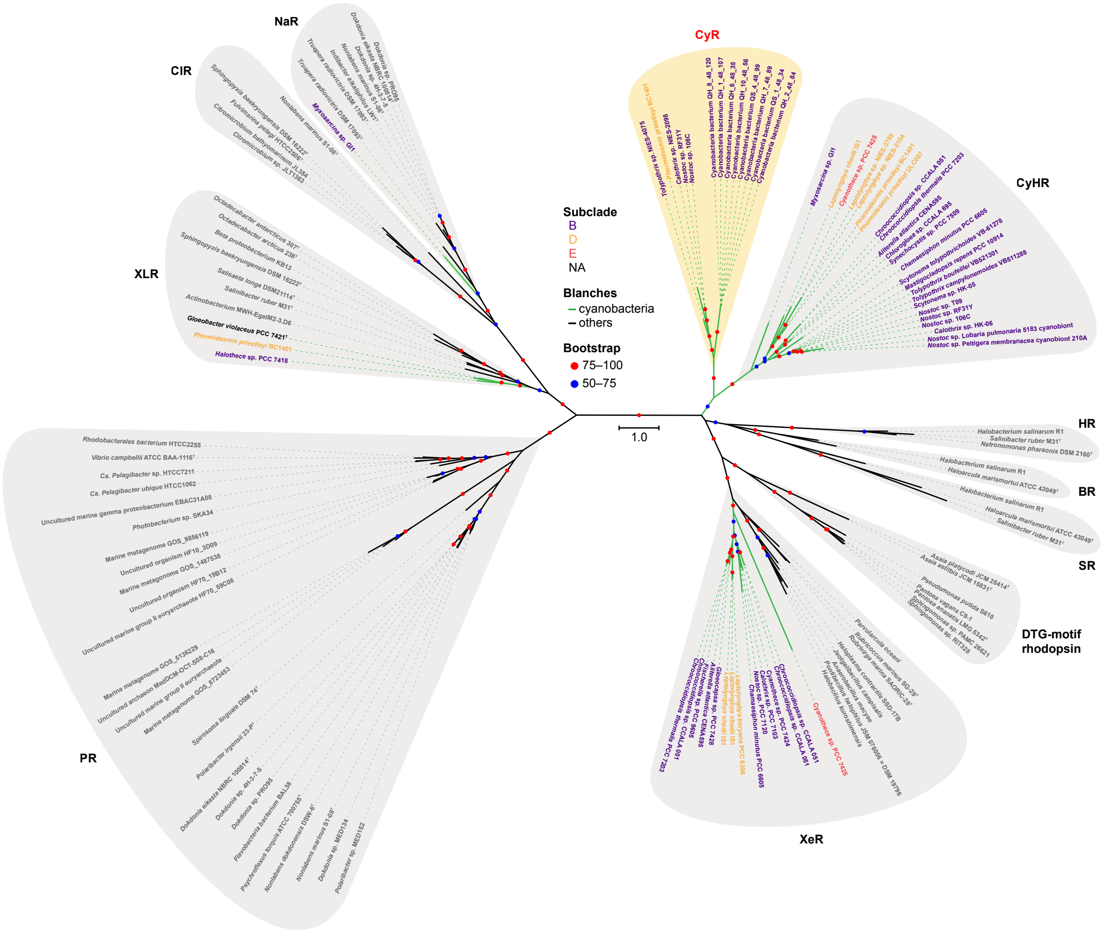

Figure S1. Phylogenetic tree of prokaryotic rhodopsins. A maximum likelihood tree of amino acid sequences of microbial rhodopsins. Bootstrap probabilities (≥50%) are indicated by colored circles. Green branches indicate cyanobacterial rhodopsins, and black branches indicate others. Strain names were colored based on phylogenetic subclades by Shih et al. 2013 [46], and strain names in gray are non-cyanobacterial strains. Rhodopsin families are indicated as follows: NaR (Na^+-pumping rhodopsin), ClR (Cl^--pumping rhodopsin), XLR (xanthorhodopsin-like rhodopsin), PR (proteorhodopsin), XeR (xenorhodopsin), DTG-motif rhodopsin, SR (sensory rhodopsin-I and sensory rhodopsin-II), BR (bacteriorhodopsin), HR (halorhodopsin), CyHR (cyanobacterial halorhodopsin), and a novel cyanobacteria-specific clade (cyanorhodopsin; CyR). The scale bar represents substitutions per site.

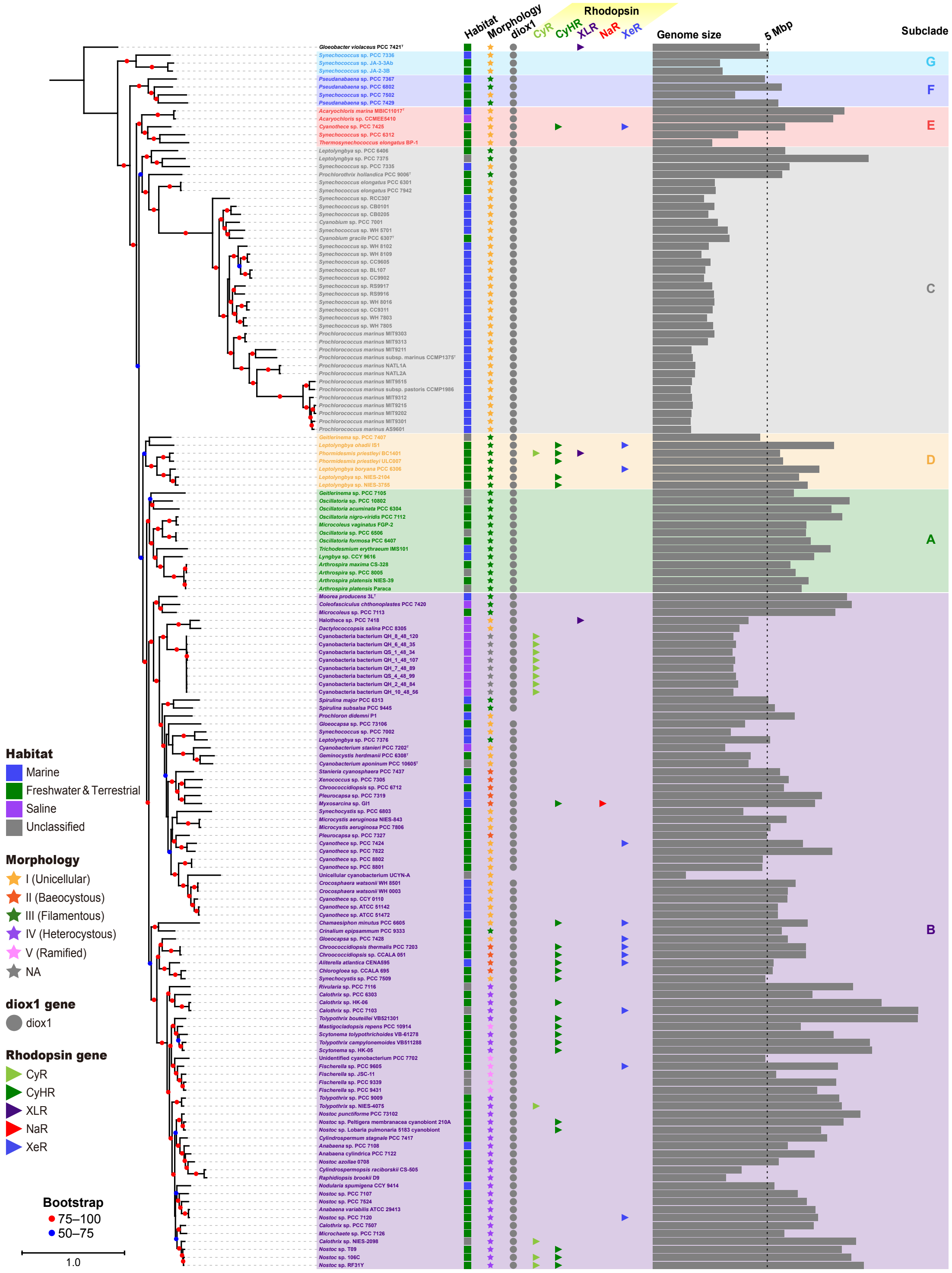

Figure S2. Genome phylogenetic tree of cyanobacteria. The tree was computed by maximum likelihood estimation based on conserved phylogenetic marker proteins (120 ubiquitous single-copy proteins). Bootstrap probabilities (≥50%) are indicated by colored circles. Strain names were colored based on phylogenetic subclades by Shih et al. 2013 [46]. Habitats and morphology of each strain are indicated by squares and stars in different colors, respectively. Morphology classifications is based on

Rippka et al. 1979 [47]. Presence of *diox1* are indicated by gray circles, and that of rhodopsin genes are indicated by right pointing triangles in different colors. Note that the *blh* genes, which encode other enzyme involved in the retinal synthesis were not detected from any genomes included in the figure. The scale bar represents substitutions per site.

```
N2098R    ---------- ---------- MTQ------- ---------- ---------- ---------- -----F---- WLWVGFIGMV
B1401R    ---------- ---------- MTAT------ ---------- ---------- ---------- --TQ-I---- WLWIAFIGMA
N4075R    ---------- ---------- MTQL------ ---------- ---------- ---------- --AL-I---- GLWIGFIGMV
MrHR      ---------- ---------- MTQ------- ---------- ---------- ---------- ------A--- WLWIGVISMA
SyHR      ---------- ---------- MAQ------- ---------- ---------- ---------- ------I--- WLWIGVIGMA
BR        MLELLPTAVE GVSQAQITGR PEWIWLA--L G--------- ---------- ---------- ---------- -----TALMG
PR        ---------- ---------- MKLLLI--LG SVIALPT--- ---------F AAGGGDLDAS DY-----TGV SFWLVTAALL
GR        ---------- ---------- MLMTVFSS-- APELALLGST FAQVDPSNLS VSDSLTYGQF NL--VYN--- AFSFAIAAMF
ASR       ---------- ---------- MNL------- ---------- ---------- ---------- --ES-L---- LHWIYVAGMT

                                               56                                                    85  89
                                              (49)                                                  (74)(78)
N2098R    IGCIYF-GMK ASAMRRREGM EFPLESFFIT LWAAALYLTM ILG-ETVTPI ---------- --NGQTV-FW GRYIDWVVTT
B1401R    LGSVAF-GLK AAGQRHREGM EFSMVSFFIT LWAATMYLSM ILS-ETVLFD ---------- FKGQHIL-FW GRYLDWVITT
N4075R    IGAVIF-GQK AVAMRRKEGM EFPLKSFFIV LWAGALYLTM ILG-ETVTPV ---------- --KDQTV-FW GRYVDWVVTT
MrHR      LGSVFF-GFG AHNAKNERWQ ILYTLNFFIC LIAAGLYLAM ALG-LGVNVI ---------- --NGRPT-YW VRFVTWFCST
SyHR      LGSIFF-GIG AHNAKNERWK ILFTINFFIC AIATGLYLSM ALG-QGRSVI ---------- --AGRPT-VW VRYITWFLST
BR        LGTLYFL-VK GMGVSDPDAK KFYAITTLVP AIAFTMYLSM LLG-YGLTMV -------PFG -GEQNPI-YW ARYADWLFTT
PR        ASTVFF-FV- ERDRVSAKWK TSLTVSGLVT GIAFWHYMYM RG----VW-- ------IE-- --TGDSP-TV FRYIDWLLTV
GR        ASALFF-FS- AQALVGQRYR LALLVSAIVV SIAGYHYFRI FNSWD-AAYV LENGVYSL-- --TSEKFNDA YRYVDWLLTV
ASR       IGALHF-WSL SRNPRGVP-Q YEYLVAMFIP IWSGLAYMAM AID-QGKVEA ---------- --AGQIA-HY ARYIDWMVTT

              96
             (85)
N2098R    PLLLMELGVI AGL----RP- ---KLIAGVM GADIFMIVTG FIGAVEA-P- P-YNYLWWLI STGSFLAILG SLLT-EYSAS
B1401R    PLLLLDLGII SGV----RP- ---KLILGVI GADVFMIVTG FIATLEG-A- P-TNYLWYAI SCGAFLAILG ALFT-EFSDT
N4075R    PVLLLDLGVL AGL----RP- ---KLIAGVI AADIFMILTG LVATLEA-P- P-TSYLWYII SCGAFIAILA SLLT-EFTAS
MrHR      PLLLLDLTFL GRT----SL- ---PLTGSLL GANAYMLVTG FVATVTP--K P-MSYIWYIV SCAAYLAIVY LLAQ-PYRIA
SyHR      PLLILDLTFL GKT----SL- ---PITASLL GANAYMIATG FVATISA-DR T-IGHIWYVV SCFAFLATVY LLVN-QYRKQ
BR        PLLLLDLALL VDA----DQ- ---GTILALV GADGIMIGTG LVGALTKV-Y S-YRFVWWAI STAAMLYILY VLFF-GFTSK
PR        PLLICEFYLI LAA----ATN VAGSLFKKLL VGSLVMLVFG YMGEAG-I-- M-AAWPAFII GCLAWVYMIY ELWAGEGKSA
GR        PLLLVETVAV LTL----PAK EARPLLIKLT VASVLMIATG YPGEISDD-I T-TRIIWGTV STIPFAYILY VLWV-ELSRS
ASR       PLLLLSLSWT AMQFIKKDW- ---TLIGFLM STQIVVITSG LIADLSE-R- DWVRYLWYIC GVCAFLIILW GIWN-PLRAK

                                              194         204    212 216
                                             (182)       (192)    (200)(204)
N2098R    AKRRNGRINS LFQTLRNILI VLWICYPIVW ILGAEGFHVI SVG-WETLCY SVLDVCAKVG FGFVVVSAGN E---------
B1401R    ARLRDGKVKQ LFLRLRNILT VLWFGYPIVW LLGTEGLQTI NVG-FETACY AILDLCAKVG FGLVLVSAGR D---------
N4075R    AARRNVRVNN LFLKLRNYLI VLWICYPIVW LLGAEAFKII PTG-VEVVIY AIIDIAAKVG FGLILTSAAP EILAQASNSE
MrHR      AERKHPRSKQ AFRTLVTVHL VLWTLYPIVW ILSPEGFSTF TQG-SETMFY TLLDIASKVG FGFLSLNTLH TL--------
SyHR      AERNYPQAKK VFRKLLSVHL VLWTLYPIVW LLGNTGFNAV NQG-TETMFY TILDITSKVG FGFLSLNSMH TL--------
BR        AESMRPEVAS TFKVLRNVTV VLWSAYPVVW LIGSEGAGIV PLN-IETLLF MVLDVSAKVG FGLILLRSRA IF--------
PR        CNTASPAVQS AYNTMMYIII FGWAIYPVGY FTGYLMGDGG SAL-NLNLIY NLADFVNKIL FGLIIWNVAV K---------
GR        LVRQPAAVQT LVRNMRWLLL LSWGVYPIAY LLPMLGVSGT SAAVGVQVGY TIADVLAKPV FGLLVFAIAL V---------
ASR       TRTQSSELAN LYDKLVTYFT VLWIGYPIVW IIGPSGFGWI NQT-IDTFLF CLLPFFSKVG FSFLDLHGLR NL--------

N2098R     ---------- --TLA--Q-- ---------A -SNSDRI-ME TVHSYMQSEE -REQSPYR-- ---------- ---------
B1401R     ---------- --VLS--E-- ---------A -SSGERR-QQ VA-----EAY LNEPTSRR-- ---------- ---------
N4075R     SFMEAVHSYM GKGER--E-- ---------R -DRSYTP--- ---------- ---------- ---------- ---------
MrHR       ---------- ---EQ--A-- ---------T E-PARET-HL ---------- ---SY----- ---------- ---------
SyHR       ---------- ---EK--N-- ---------T E-SVSSY-ES ---------- ---STI---- ---------- ---------
BR         ---------- ---GE--A-- ---------E -APEPSA--- ------G--- --DGAAATSD ---------- ---------
PR         --E------- ---SSN---- ---------- -------A-- ---------- ---------- ---------- ---------
GR         --K------- ---TKADQES SEPHAAIGA- A-ANKS-GGS ---------- ---LIS---- ---------- ---------
ASR        ---------- ---ND--S-- ---------R -QTTG-D-RF -AE------- --NTLQF--- VENITLFANS RRQQSRRRV
```

Figure S3. Sequence alignment of rhodopsins. The accessions and rhodopsin families are as follows: N2098R (BAY09002.1, CyR), B1401R (WP_074382570.1, CyR), N4075R (GAX43141.1, CyR), MrHR (WP_017314391.1, CyHR), SyHR (WP_009632765.1, CyHR), BR (CAP14056.1, BR), PR (AAG10475.1, PR), GR (BAC88139.1, XLR), and ASR (BAB74864.1, XeR). Columns of functionally important residues are shown in bold. The numbers above the columns indicate amino acid numbers in BR, and N2098R in parentheses. Known functions are as follows: primary proton acceptor (Asp85), proton donor (Glu96), proton release group (Glu194 and Glu204), counterion (Asp212), Schiff base (Lys216). Two carboxylates, Asp (D) and Glu (E), are shown in blue, and Schiff base Lys (K) is shown in red.

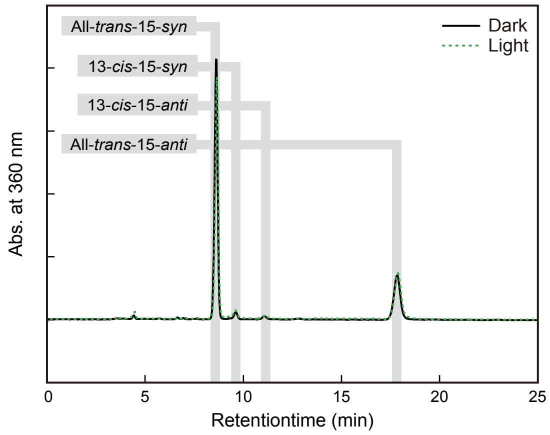

Figure S4. Retinal configuration of N2098R. HPLC patterns of retinal isomers of N2098R with (green broken line) and without (solid line) light illumination at 550 ± 10 nm for 10 min. The retinal isomer exacted as retinal oximes. Molar comparison of each retinal isomer was calculated from areas of peaks in the HPLC patterns using absorption coefficients of retinal isomers (51,600, 54,600, and 47,900 cm^{-1} M^{-1} for all-*trans* 15-*anti*, all-*trans* 15-*syn*, and 13-*cis* retinal oximes, respectively).

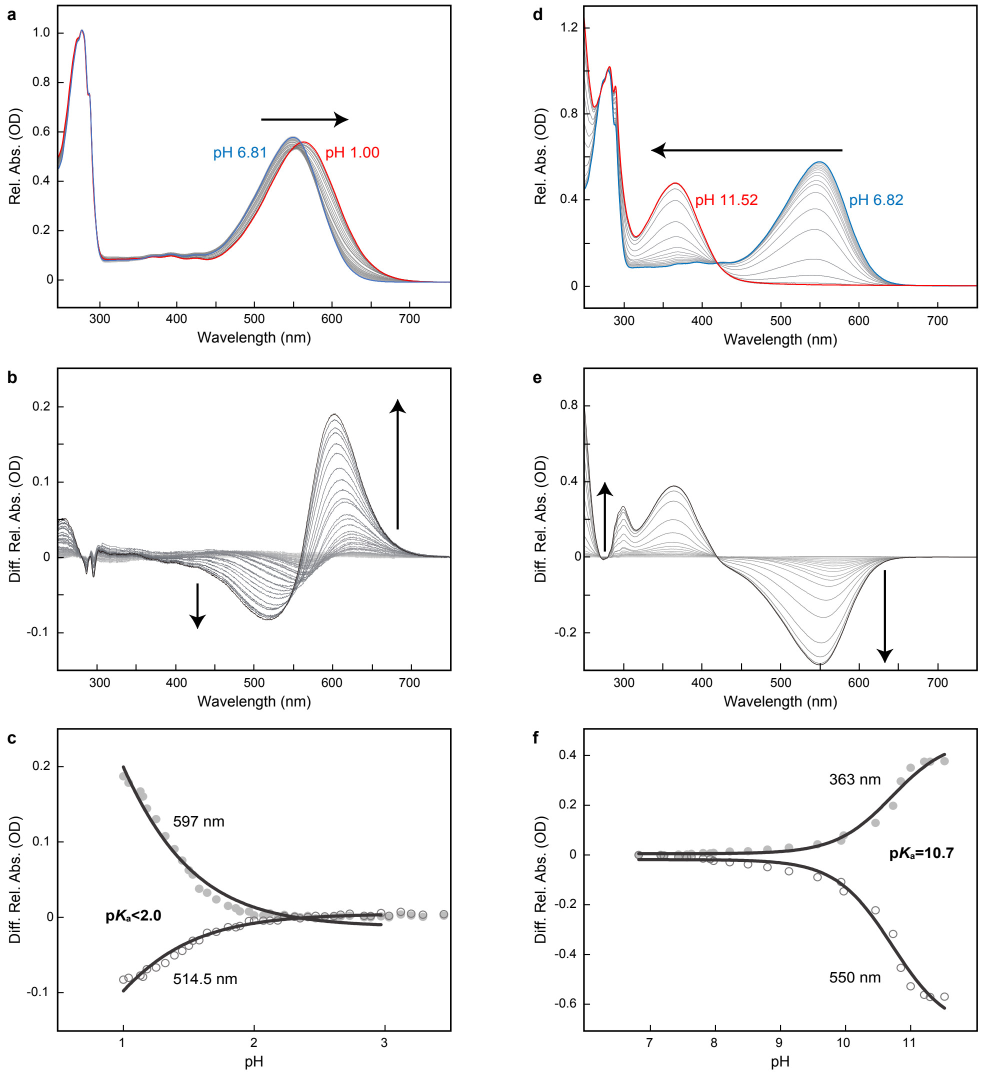

Figure S5. pH-induced spectral changes of N2098R over a spectral range of 250–750 nm. Sample was suspended in a seven mix buffer with 50 mM NaCl and 0.05% DDM. (**a**) Absorption spectra at acidic pH from 6.81 (blue line) to 1.00 (red line), where the pH was adjusted to the desired value by adding 1N HCl. Absorption spectra changes were indicated gradation line from light gray (pH 6.81) to black (pH 1.00). (**b**) Difference spectra at acidic pH (6.81–1.00). Spectrum at pH 6.81 was subtracted from each spectrum and is described as a baseline. (**c**) Estimation of pK_a value of Asp74 in N2098R. Absorption differences at 514.5 nm (opened circles) and 597 nm (closed circles) were plotted against pH values. The data ranging from pH 1.00 to 2.97 were analyzed by using the Henderson-Hasselbalch equation with a single pK_a (solid lines). (**d**) Absorption spectra at alkaline pH from 6.82 (blue line) to 11.52 (red line), where the pH was adjusted to the desired value by adding 1N NaOH. Absorption spectra changes were indicated gradation line from light gray (pH 6.82) to black (pH 11.52). (**e**) Difference spectra at alkaline pH (6.82–11.52). The spectrum at

pH 6.82 was subtracted from each spectrum and is described as a baseline. (**f**) Estimation of the pK_a value of Lys204 in N2098R. Absorption differences at 363 nm (closed circles) and 550 nm (opened circles) were plotted against pH values. The data ranging from pH 6.82 to 11.52 were analyzed by using the Henderson-Hasselbalch equation with a single pK_a (solid lines).

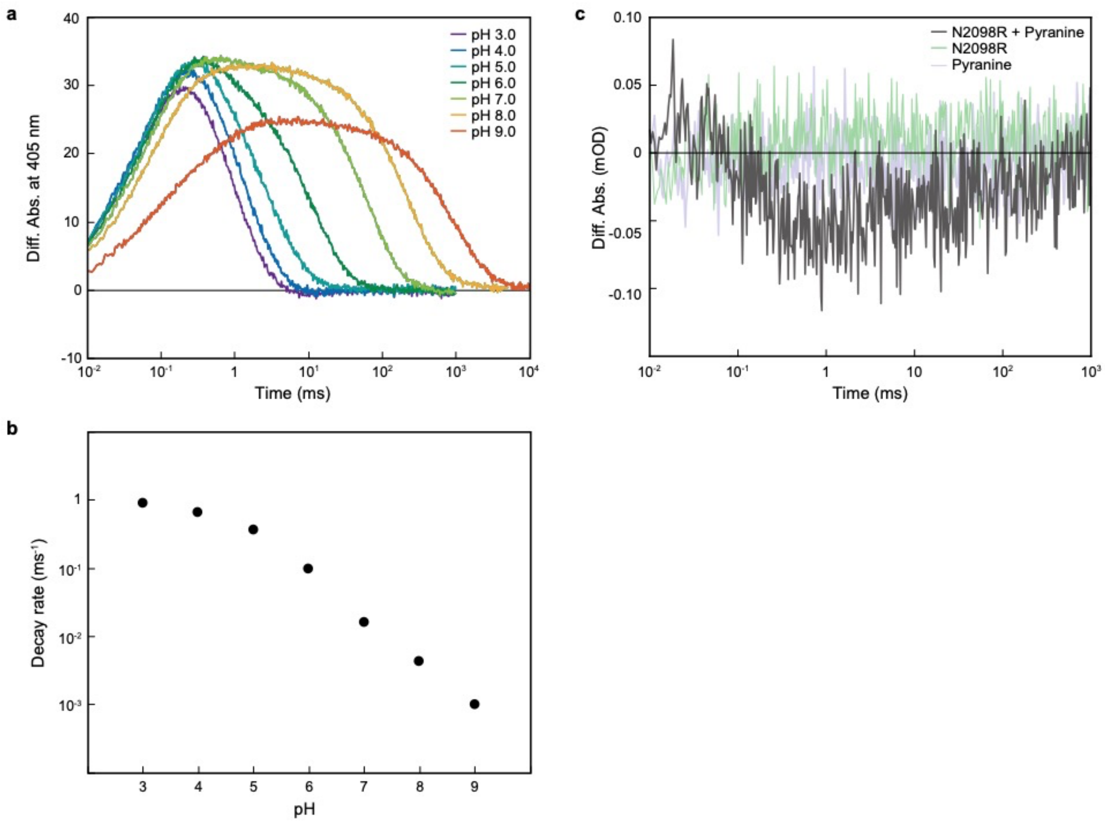

Figure S6. Flash analyses of N2098R. M-decay of N2098R at varying pH under room temperature. pH-dependency on curve at M-intermediate (405 nm) (**a**) and estimated M-decay rate constant at varying pH (**b**). (**c**) Flash-induced kinetic data of N2098R and pyranine (gray line), only N2098R (green line), and only pyranine (purple line). The gray line represents the absorption changes of pyranine monitored at 450 nm.

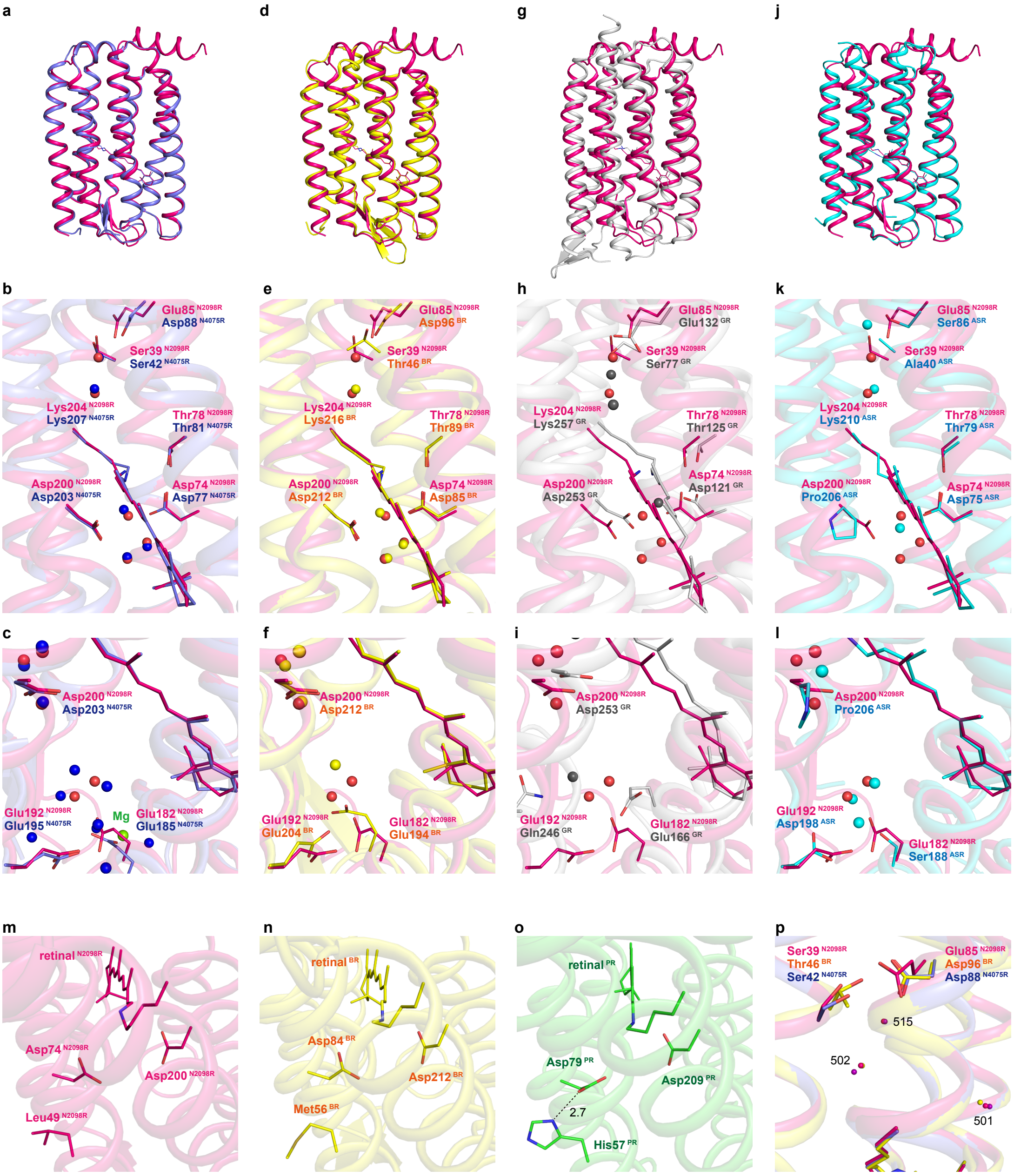

Figure S7. Structural comparison between N2098R (magenta; PDB code 6LM0), N4075R (blue; 6LM1), BR (yellow; 1C3W), GR (gray; 6NWD), ASR (cyan; 1XIO), and PR (green; 4JQ6). Overall structural comparison between N2098R (magenta) and N4075R (blue), viewed parallel to the membrane (**a**), and structural comparison between N2098R (magenta) and N4075R (blue) of the retinal binding region (**b**), and proton release region (**c**). Overall structural comparison between N2098R (magenta) and BR (yellow), viewed parallel to the membrane (**d**), and structural comparison between N2098R (magenta) and BR (yellow) of the retinal binding region (**e**), and proton release region (**f**). Overall structural comparison between N2098R (magenta) and ASR (cyan), viewed parallel to the membrane (**g**),

and structural comparison between N2098R (magenta) and GR (gray) of the retinal binding region (**h**), and proton release region (**i**). Overall structural comparison between N2098R (magenta) and ASR (cyan), viewed parallel to the membrane (**j**), and structural comparison between N2098R (magenta) and ASR (cyan) of the retinal binding region (**k**), and proton release region (**l**). Structure of the near the primary proton acceptor in N2098R (**m**), BR (**n**), and PR (**o**). (**p**) Structure comparison around the proton donor between N2098R (Glu85), BR (Asp96), and N4075R (Asp88).

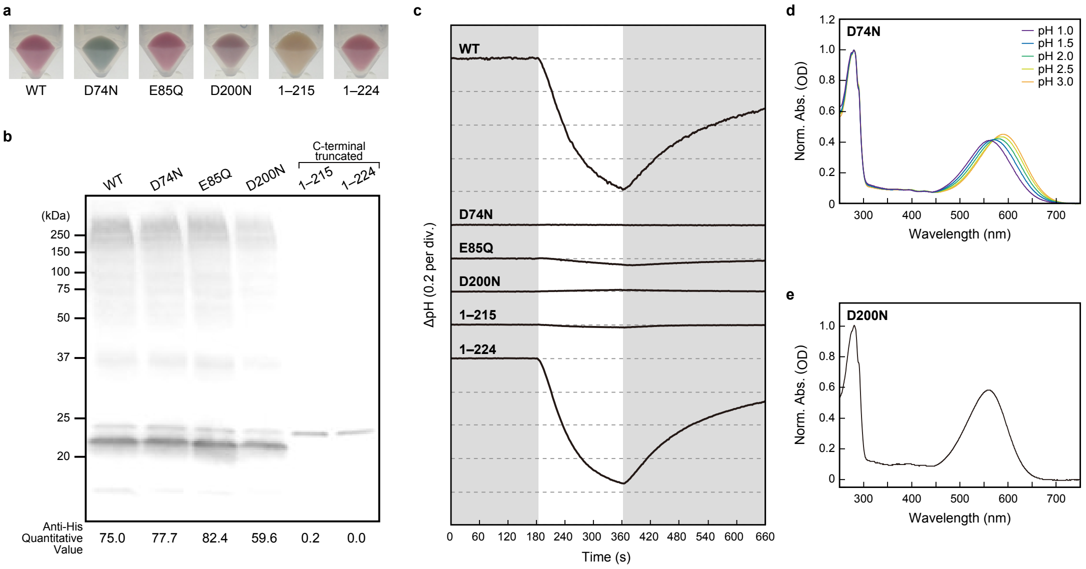

Figure S8. Mutation analyses of N2098R. (**a**) The pellet color of mutants. (**b**) Detection of protein expression of mutants by western blots using an anti-His-tag antibody. These proteins were expressed in *E. coli* cells with a His-tag at the C-terminal except 1–215 and 1–224 truncated proteins inserted the stop codon before a His-tag region. The monomer-band of mutants (around 22 kDa) were quantified using ImageJ software. (**c**) Light-induced pH changes of N2098R mutants-expressing *E. coli* cell suspensions at 4°C. pH changes of *E. coli* cells expressing N2098R WT, D74N, E85Q, D200N, 1–215, and 1–224. All measurements were performed under the dark condition (gray background) and illuminated at 520 ± 10 nm for 3 min (white background). (d) UV–Vis spectra of D74N mutant under the low pH. (e) UV–Vis spectra of D200N mutant.

Table S1. Rhodopsin distribution in cyanobacterial lineage.

Clade	Subclade	Strain
Xanthorhodopsin-like rhodopsin (XLR)	B	*Halothece* sp. PCC 7418
	D	*Phormidesmis priestleyi* BC1401
	NA	*Gloeobacter violaceus* PCC 7421 (GR)
Xenorhodopsin (XeR)	B	*Aliterella atlantica* CENA595
		Calothrix sp. PCC 7103
		Chamaesiphon minutus PCC 6605
		Chroococcidiopsis sp. CCALA 051
		Chroococcidiopsis thermalis PCC 7203
		Cyanothece sp. PCC 7424
		Fischerella sp. PCC 9605
		Gloeocapsa sp. PCC 7428
		Nostoc sp. PCC 7120
	D	*Leptolyngbya boryana* PCC 6306
		Leptolyngbya ohadii IS1
	E	*Cyanothece* sp. PCC 7425
Na^+-pumping rhodopsin (NaR)	B	*Myxosarcina* sp. GI1
Cyanobacterial halorhodopsin (CyHR)	B	*Aliterella atlantica* CENA595
		Calothrix sp. HK-06
		Chamaesiphon minutus PCC 6605
		Chlorogloea sp. CCALA 695
		Chroococcidiopsis sp. CCALA 051
		Chroococcidiopsis thermalis PCC 7203
		Mastigocladopsis repens PCC 10914
		Myxosarcina sp. GI1
		Nostoc sp. 106C
		Nostoc sp. Lobaria pulmonaria 5183 cyanobiont
		Nostoc sp. Peltigera membranacea cyanobiont 210A
		Nostoc sp. RF31Y
		Nostoc sp. T09
		Scytonema sp. HK-05
		Scytonema tolypothrichoides VB-61278
		Synechocystis sp. PCC 7509
		Tolypothrix bouteillei VB521301
		Tolypothrix campylonemoides VB511288
	D	*Leptolyngbya ohadii* IS1
		Leptolyngbya sp. NIES-2104
		Leptolyngbya sp. NIES-3755
		Phormidesmis priestleyi BC1401
		Phormidesmis priestleyi ULC007
	E	*Cyanothece* sp. PCC 7425
Cyanorhodopsin (CyR)	B	*Calothrix* sp. NIES-2098
		Cyanobacteria bacterium QH_1_48_107
		Cyanobacteria bacterium QH_7_48_89
		Cyanobacteria bacterium QH_2_48_84
		Cyanobacteria bacterium QH_10_48_56
		Cyanobacteria bacterium QS_1_48_34
		Cyanobacteria bacterium QH_6_48_35
		Cyanobacteria bacterium QS_4_48_99
		Cyanobacteria bacterium QH_8_48_120
		Nostoc sp. 106C
		Nostoc sp. RF31Y
		Tolypothrix sp. NIES-4075
	D	*Phormidesmis priestleyi* BC1401

Table S2. X-ray data collection, phasing and refinement statistics.

	N2098R	N4075R
PDB ID	6LM0	6LM1
No. of Crystals for refinement	214	1
Data collection		
Space group	*C222₁*	*C222₁*
Cell dimensions		
a, b, c (Å)	64.5, 107.6, 224.5	107.9, 129.7, 127.6
α, β, γ(°)	90.0, 90.0, 90.0	90.0, 90.0, 90.0
Wavelength (Å)	1.0	1.0
Resolution (Å)	44.48 - 2.65 (2.745 - 2.65)	45.48 - 1.9 (1.968 - 1.9)
Total reflections	1678894 (170204)	141176 (14040)
Unique reflections	23176 (2289)	70588 (7020)
Redundancy	72.4 (74.4)	2.0 (2.0)
Completeness (%)	99.9 (99.8)	99.9 (99.9)
$I/\sigma(I)$	10.8 (1.8)	18.3 (3.0)
R-merge (%)	72.7 (727.7)	20.9 (22.8)
R-meas (%)	73.1 (732.2)	29.6 (32.3)
$CC_{1/2}$ (%)	99.3 (63.3)	100 (90.2)
Refinement		
R_{work} (%)	22.7	17.0
R_{free} (%)	27.4	20.6
r.m.s.d. bond lengths (Å)	0.002	0.007
r.m.s.d. bond angles (°)	0.65	0.74
Number of water molecules	59	189
Average B factor (Å^2)		
all	41.3	36.9
protein	41.4	34.5
ligand	37.5	52.2
solvent	40.4	50.2
Ramachandran plot		
Most favored regions (%)	97.3	99.1
outliers (%)	0	0

Statistics for the highest-resolution shell are shown in parentheses.

Table S3. Comparing the root-mean-square deviation (RMSD) with N2098R and N4075R.

	N2098R	N4075R
N4075R	0.733 Å	–
Bacteriorhodopsin (BR; 1C3W)	0.994 Å	1.100 Å
Anabaena sensory rhodopsin (ASR; 1XIO)	1.404 Å	1.257 Å
Proteorhodopsin (PR; 4JQ6)	1.717 Å	1.637 Å
Xanthorhodopsin (XR; 3DDL)	1.901 Å	2.176 Å
Gloeobacter rhodopsin (GR; 6NWD)	1.910 Å	2.190 Å

Supplementary References

1. Parks, D. H., Imelfort, M., Skennerton, C. T., Hugenholtz, P. & Tyson, G. W. CheckM: assessing the quality of microbial genomes recovered from isolates, single cells, and metagenomes. *Genome Res.* **25**, 1043–1055 (2015).
2. Eddy, S. R. Accelerated profile HMM searches. *PLoS Comput. Biol.* **7**, e1002195 (2011).
3. Stamatakis, A. RAxML version 8: a tool for phylogenetic analysis and post-analysis of large phylogenies. *Bioinformatics* **30**, 1312–1313 (2014).
4. Parks, D. H. *et al.* A standardized bacterial taxonomy based on genome phylogeny substantially revises the tree of life. *Nat. Biotechnol.* **36**, 996–1004 (2018).
5. Letunic, I. & Bork, P. Interactive tree of life (iTOL) v3: an online tool for the display and annotation of phylogenetic and other trees. *Nucleic Acids Res.* **44**, W242–W245 (2016).
6. Sudo, Y. & Yoshizawa, S. Functional and photochemical characterization of a light-driven proton pump from the Gammaproteobacterium *Pantoea vagans*. *Photochem. Photobiol.* **92**, 420–427 (2016).
7. Groenendljk, G. W. T., De Grip, W. J. & Daemen, F. J. M. Quantitative determination of retinals with complete retention of their geometric configuration. *Biochim. Biophys. Acta (BBA)/Lipids Lipid Metab.* **617**, 430–438 (1980).
8. Groenendijk, G. W. T., De Grip, W. J. & Daemen, F. J. M. Identification and characterization of syn- and anti-isomers of retinaloximes. *Anal. Biochem.* **99**, 304–310 (1979).
9. Trehan, A. *et al.* On retention of chromophore configuration of rhodopsin isomers derived from three dicis retinal isomers. *Bioorg. Chem.* **18**, 30–40 (1990).
10. Matsuyama, T., Yamashita, T., Imamoto, Y. & Shichida, Y. Photochemical properties of mammalian melanopsin. *Biochemistry* **51**, 5454–5462 (2012).
11. Suzuki, D. *et al.* Effects of chloride ion binding on the photochemical properties of *Salinibacter* sensory rhodopsin I. *J. Mol. Biol.* **392**, 48–62 (2009).

12. Inoue, S. *et al.* Spectroscopic characteristics of *Rubricoccus marinus* xenorhodopsin (*Rm*XeR) and a putative model for its inward H^+ transport mechanism. *Phys. Chem. Chem. Phys.* **20**, 3172–3183 (2018).
13. Hosaka, T. *et al.* Structural mechanism for light-driven transport by a new type of chloride ion pump, nonlabens marinus rhodopsin-3. *J. Biol. Chem.* **291**, 17488–17495 (2016).
14. Furuse, M. *et al.* Structural basis for the slow photocycle and late proton release in *Acetabularia* rhodopsin I from the marine plant *Acetabularia acetabulum*. *Acta Crystallogr. Sect. D Biol. Crystallogr.* **71**, 2203–2216 (2015).
15. Shimono, K. *et al.* Production of functional bacteriorhodopsin by an *Escherichia coli* cell-free protein synthesis system supplemented with steroid detergent and lipid. *Protein Sci.* **18**, 2160–2171 (2009).
16. Kigawa, T. *et al.* Preparation of *Escherichia coli* cell extract for highly productive cell-free protein expression. *J. Struct. Funct. Genomics* **5**, 63–68 (2004).
17. Hato, M., Hosaka, T., Tanabe, H., Kitsunai, T. & Yokoyama, S. A new manual dispensing system for in meso membrane protein crystallization with using a stepping motor-based dispenser. *J. Struct. Funct. Genomics* **15**, 165–171 (2014).
18. Hirata, K. *et al.* Achievement of protein micro-crystallography at SPring-8 beamline BL32XU. *J. Phys. Conf. Ser.* **425**, 012002 (2013).
19. Hirata, K. *et al. ZOO*: an automatic data-collection system for high-throughput structure analysis in protein microcrystallography. *Acta Crystallogr. Sect. D Struct. Biol.* **75**, 138–150 (2019).
20. Yamashita, K., Hirata, K. & Yamamoto, M. *KAMO*: towards automated data processing for microcrystals. *Acta Crystallogr. Sect. D Struct. Biol.* **74**, 441–449 (2018).
21. Kabsch, W. *XDS*. *Acta Crystallogr. Sect. D Biol. Crystallogr.* **66**, 125–132 (2010).
22. Evans, G., Axford, D., Waterman, D. & Owen, R. L. Macromolecular microcrystallography. *Crystallogr. Rev.* **17**, 105–142 (2011).
23. Foadi, J. *et al.* Clustering procedures for the optimal selection of data sets from multiple crystals in macromolecular crystallography. *Acta Crystallogr. Sect. D*

Biol. Crystallogr. **69**, 1617–1632 (2013).

24. McCoy, A. J. *et al. Phaser* crystallographic software. *J. Appl. Crystallogr.* **40**, 658–674 (2007).
25. Adams, P. D. *et al. PHENIX*: a comprehensive python-based system for macromolecular structure solution. *Acta Crystallogr. Sect. D Biol. Crystallogr.* **66**, 213–221 (2010).
26. Luecke, H., Schobert, B., Richter, H. T., Cartailler, J. P. & Lanyi, J. K. Structure of bacteriorhodopsin at 1.55 Å resolution. *J. Mol. Biol.* **291**, 899–911 (1999).
27. Emsley, P. & Cowtan, K. *Coot*: Model-building tools for molecular graphics. *Acta Crystallogr. Sect. D Biol. Crystallogr.* **60**, 2126–2132 (2004).
28. Balashov, S. P. Protonation reactions and their coupling in bacteriorhodopsin. *Biochimica et Biophysica Acta - Bioenergetics* **1460**, 75–94 (2000).
29. Lanyi, J. K. Proton transfers in the bacteriorhodopsin photocycle. *Biochim. Biophys. Acta - Bioenerg.* **1757**, 1012–1018 (2006).
30. Subramaniam, S., Marti, T. & Khorana, H. G. Protonation state of Asp (Glu)-85 regulates the purple-to-blue transition in bacteriorhodopsin mutants Arg-82 → Ala and Asp-85 → Glu: the blue form is inactive in proton translocation. *Proc. Natl. Acad. Sci. U. S. A.* **87**, 1013–1017 (1990).
31. Sharaabi, Y., Brumfeld, V. & Sheves, M. Binding of anions to proteorhodopsin affects the Asp97 pK_a. *Biochemistry* **49**, 4457–4465 (2010).
32. Druckmann, S., Ottolenghi, M., Pande, A., Pande, J. & Callender, R. H. Acid-base equilibrium of the Schiff base in bacteriorhodopsin. *Biochemistry* **21**, 4953–4959 (1982).
33. Imasheva, E. S., Balashov, S. P., Wang, J. M., Dioumaev, A. K. & Lanyi, J. K. Selectivity of retinal photoisomerization in proteorhodopsin is controlled by aspartic acid 227. *Biochemistry* **43**, 1648–1655 (2004).
34. Choi, A. R., Shi, L., Brown, L. S. & Jung, K. H. Cyanobacterial light-driven proton pump, *Gloeobacter* rhodopsin: complementarity between rhodopsin-based energy production and photosynthesis. *PLoS One* **9**, e110643 (2014).
35. Kano, K. & Fendler, J. H. Pyranine as a sensitive pH probe for liposome interiors and surfaces. pH gradients across phospholipid vesicles. *BBA - Biomembr.* **509**, 289–299 (1978).

36. Kojima, K. *et al.* Vectorial proton transport mechanism of RxR, a phylogenetically distinct and thermally stable microbial rhodopsin. *Sci. Rep.* **10**, 1–12 (2020).
37. Grzesiek, S. & Dencher, N. A. Time-course and stoichiometry of light-induced proton release and uptake during the photocycle of bacteriorhodopsin. *FEBS Lett.* **208**, 337–342 (1986).
38. Dioumaev, A. K. *et al.* Proton transfers in the photochemical reaction cycle of proteorhodopsin. *Biochemistry* **41**, 5348–5358 (2002).
39. Needleman, R. *et al.* Properties of Asp212 → Asn bacteriorhodopsin suggest that Asp212 and Asp85 both participate in a acounterion and proton acceptor complex near the Schiff base. **266**, 11478–11484 (1991).
40. Moltke, S., Krebs, M. P., Mollaaghababa, R., Khorana, H. G. & Heyn, M. P. Intramolecular charge transfer in the Bacteriorhodopsin mutants Asp85→Asn and Asp2l2→Asn : Effects of pH and anions. *Biophys. J.* **69**, 2074–2083 (1995).
41. Hempelmann, F. *et al.* His75-Asp97 cluster in green proteorhodopsin. *J. Am. Chem. Soc.* **133**, 4645–4654 (2011).
42. Cai, K. *et al.* Single-cysteine substitution mutants at amino acid positions 306–321 in rhodopsin, the sequence between the cytoplasmic end of helix VII and the palmitoylation sites: sulfhydryl reactivity and transducin activation reveal a tertiary structure. *Biochemistry* **38**, 7925–7930 (1999).
43. Ernst, O. P. *et al.* Mutation of the fourth cytoplasmic loop of rhodopsin affects binding of transducin and peptides derived from the carboxyl-terminal sequences of transducin α and γ subunits. *J. Biol. Chem.* **275**, 1937–1943 (2000).
44. Krishna, A. G., Menon, S. T., Terry, T. J. & Sakmar, T. P. Evidence that helix 8 of rhodopsin acts as a membrane-dependent conformational switch. *Biochemistry* **41**, 8298–8309 (2002).
45. Fritze, O. *et al.* Role of the conserved NPxxY(x)$_{5,6}$ motif in the rhodopsin ground state and during activation. *Proc. Natl. Acad. Sci. U. S. A.* **100**, 2290–2295 (2003).
46. Shih, P. M. *et al.* Improving the coverage of the cyanobacterial phylum using diversity-driven genome sequencing. *Proc. Natl. Acad. Sci. U. S. A.* **110**, 1053–1058 (2013).

47. Rippka, R., Deruelles, J., Waterbury, J. B., Herdman, M. & Stainer, R. Generic assignments, strain histories and properties of pure cultures of cyanobacteria. *J. Gen. Microbiol.* **111**, 1–61 (1979).
